# Airway epithelial cell-specific deletion of EGFR modulates mucoinflammatory features of cystic fibrosis-like lung disease in mice

**DOI:** 10.3389/fimmu.2025.1493950

**Published:** 2025-05-08

**Authors:** Ishita Choudhary, Kshitiz Paudel, Rahul Kumar, Amit Sharma, Sonika Patial, Yogesh Saini

**Affiliations:** ^1^ Department of Population Health and Pathobiology, College of Veterinary Medicine, North Carolina State University, Raleigh, NC, United States; ^2^ Division of Translational Toxicology, National Institute of Environmental Health Sciences, Research Triangle Park, Durham, NC, United States

**Keywords:** EGFR, cystic fibrosis, *Scnn1b*-Tg+, airway epithelium, mucus obstruction

## Abstract

Mucoinflammatory lung disease in cystic fibrosis (CF) is characterized by airway surface liquid (ASL) layer dehydration and mucins hyperconcentration, which leads to airway obstruction, inflammation, bronchiectasis, and increased susceptibility to recurrent bacterial infections. Epidermal growth factor receptor (EGFR) is known to regulate airway mucous cell metaplasia (MCM) and mucins expression, but the role of EGFR pathway in the pathogenesis of CF-like lung disease remains unclear. Therefore, we hypothesized that airway epithelial cell-specific deficiency of EGFR mitigates mucoinflammatory responses in *Scnn1b*-transgenic (Tg+) mice that phenocopy human CF-like lung disease. To test this hypothesis, we examined the effect of airway epithelial cell-specific EGFR deficiency on the manifestation of mucoinflammatory outcomes in Tg+ mice. The airway epithelial cell-specific EGFR-deficient wild-type (WT) mice did not exhibit any obvious structural and functional defects in the lungs. The deletion of EGFR in airway epithelial cells in Tg+ mice, however, resulted in increased recruitment of neutrophils and macrophages into the lung airspaces, which was accompanied by significantly increased bronchoalveolar lavage fluid (BALF) levels of inflammatory mediators, including KC, G-CSF, MIP-2, MIP-1α, TNF-α, and MIP-1β. Additionally, as compared with the EGFR-sufficient Tg+ mice, the airway epithelial cell-specific EGFR-deficient Tg+ mice exhibited significantly increased postnatal mortality and compromised bacterial clearance. The deletion of EGFR in the airway epithelial cells of Tg+ mice resulted in an increased degree of mucus obstruction, which was associated with an increase in MCM and MUC5B production. Some of the molecular markers of type 2 inflammation, including *Il13*, *Slc26a4*, and *Retnla*, were significantly increased in airway epithelial cell-specific EGFR-deficient Tg+ mice versus EGFR-sufficient Tg+ mice. Taken together, our data show that EGFR deletion in the airway epithelial cells compromises postnatal survival, delays bacterial clearance, and modulates inflammatory and mucus obstruction-relevant endpoints, i.e., MCM, MUC5B production, and mucus obstruction, in Tg+ mice.

## Introduction

Cystic fibrosis (CF) is an autosomal recessive genetic disorder caused by various mutations in the cystic fibrosis transmembrane conductance regulator (*CFTR*) gene, which results in reduced chloride and bicarbonate ions secretion and increased sodium ions absorption (1, 2). The resulting ionic imbalance leads to the dehydration of the airway surface liquid layer (ASL), causing mucins hyperconcentration, mucostasis, defective mucociliary clearance, airway inflammation, and recurrent bacterial infections (2, 3). Mucins hypersecretion is another key characteristic of CF, where the excessive release of mucins into the hyperconcentrated ASL results in the formation of static mucus plugs, which in turn contributes to airflow obstruction (3). IL-4Rα and epidermal growth factor receptor (EGFR) pathways are known to contribute to mucins hypersecretion via coordinated promotion of mucous cell metaplasia (MCM) (4–10). While the role of IL-4Rα signaling pathway has been investigated in mouse model of human CF-like lung disease (11), the role of EGFR signaling pathway in inducing mucins production and other inflammatory outcomes remains unclear.

EGFR, one of the four members of the receptor tyrosine kinase (RTK) superfamily, binds to a variety of ligands, including epidermal growth factor (EGF), transforming growth factor-α (TGF-α), amphiregulin (AREG), epiregulin (EREG), β-cellulin (BTC), heparin-binding EGF (HB-EGF), and epigen (EPGN) (12, 13). EGFR is critically important for embryonic development, tissue differentiation, and cellular function, and EGFR loss causes either embryonic or postnatal mortality depending on the genetic background of the mice (14–16). In normal airways, EGFR signaling constitutes a critical developmental pathway in lung epithelial cells by controlling lung development and maintaining airway homeostasis (15, 17–20). Dysregulated EGFR signaling is associated with the pathogenesis of airway hypersecretory and mucoinflammatory diseases such as asthma and COPD (12). EGFR signaling is critical for inducing MCM in animal models and for mucins expression in human airway epithelial cells in response to IL-13, viruses, TGF-α, and cigarette smoke (6, 21–24). Increased EGFR signaling, TGF-α production, and mucins (MUC5AC and MUC5B) expression have been reported in the airways of CF patients (25); however, the causative role of EGFR in CF-like lung disease has not been demonstrated, thus warranting further investigation. Accordingly, we investigated the role of airway epithelial cell-specific EGFR in the pathogenesis of mucoinflammatory lung disease in *Scnn1b*-transgenic (*Scnn1b*-Tg+) mice, a mouse model of human CF-like lung disease.

The *Scnn1b*-Tg+ (Tg+) mouse overexpresses sodium channel, non-voltage gated 1, beta subunit (*Scnn1b*) transgene in club cell secretory protein (CCSP)-expressing airway epithelial cells (26). The *Scnn1b* overexpression dictates the hyperabsorption of sodium ions into the airway epithelial cells, resulting in an osmotic gradient-driven dehydration of ASL. As a consequence, within the first week of postnatal life, the Tg+ neonates exhibit mucoinflammatory lung disease features, including MCM, mucins hypersecretion, mucus obstruction, defective mucociliary clearance, airway inflammation characterized by activated macrophages, granulocytes, and lymphocytes, and spontaneous bacterial infections (26–30). In this study, we hypothesized that airway epithelial cell-specific deficiency of EGFR mitigates mucoinflammatory responses in Tg+ mice. Towards this, we examined the effects of airway epithelial cell-specific EGFR deletion on key features of Tg+ mice, including postnatal survival, genes relevant to mucoinflammatory responses, mucus obstruction, MCM, immune cell recruitment, cytokine levels, and bacterial load. The results from this study highlight the contribution of airway epithelial cell-specific EGFR in the pathogenesis of CF-like mucoinflammatory lung disease in Tg+ mice.

## Materials and methods

### Generation of mice and animal husbandry

*Scnn1b*-Tg+ (Tg+) mice [B6N.Cg-Tg(Scgb1a1-Scnn1b)6608Bouc/J] were procured from the Jackson Laboratory (Bar Harbor, ME) and maintained at the Division of Laboratory Animal Medicine (DLAM) vivarium of Louisiana State University (LSU). Club cell-specific Cre recombinase (CCSP-Cre^+^) mice were provided by Dr. Francesco J. DeMayo (NIEHS, North Carolina (NC)), and *Egfr* floxed (*Egfr* ^fl/fl^) mice were provided by Dr. David Threadgill (Texas A&M University, Texas). All three mice strains were interbred to generate various parental genotypes. EGFR-sufficient Tg+ (CCSP-Cre^-^/*Egfr* ^fl/fl^/Tg+) and airway epithelial cell-specific EGFR-deficient Tg+ (CCSP-Cre^+^/*Egfr* ^fl/fl^/Tg+) and their wild-type (WT) counterparts, i.e., CCSP-Cre^-^/*Egfr* ^fl/fl^/WT and CCSP-Cre^+^/*Egfr* ^fl/fl^/WT, were generated by reciprocal crosses between CCSP-Cre^+^/*Egfr* ^fl/fl^/WT and CCSP-Cre^-^/*Egfr* ^fl/fl^/Tg+ (or CCSP-Cre^-^/*Egfr* ^fl/fl^/WT and CCSP-Cre^+^/*Egfr* ^fl/fl^/Tg+) parental genotypes. The CCSP-Cre^+^/*Egfr* ^fl/fl^/Tg+ mice are expected to have EGFR deficiency only in the CCSP+ cells. However, our previous study employing CCSP-Cre mice (31) have shown that the CCSP-Cre^+^ causes recombination in almost all the airway epithelial cells. Therefore, we will be using the term “airway epithelial cell-specific EGFR-deficient mice” and not “Club cell-specific EGFR deficient mice”. Genotypes of all four experimental groups were confirmed with polymerase chain reaction (PCR). Nucleotide sequences of primers used for genotyping are included in **Supplementary Table 1**. Mice were housed in hot-washed and individually ventilated cages on 12h day/night cycle at DLAM vivarium of LSU. Mice were provided with a regular diet and water *ad libitum*. All the animal experiments were approved by the Institutional Animal Care and Use Committee (IACUC) of Louisiana State University.

### Bronchoalveolar lavage fluid analyses and tissue collection

Juvenile mice (Postnatal day 21, PND21) were anesthetized via intraperitoneal (IP) injection of 2,2,2-tribromoethanol (Millipore Sigma, Burlington, MA). The left main stem bronchus was ligated, and the right lung lobes were aseptically lavaged with a body weight-adjusted volume of Dulbecco’s Phosphate Buffered Saline (DPBS) (Corning, Manassas, VA). A fraction of the harvested bronchoalveolar lavage fluid (BALF) was used for the estimation of colony forming units (CFUs), and the remaining BALF was centrifuged at 4°C for 500 x *g* for 5 min. Cell-free BALF supernatant was collected and stored at -80°C for the total protein, dsDNA, and cytokine analyses. Cell pellets were resuspended in 250 µl of DPBS and used for total and differential cell counts estimation as described previously (32). Lavaged right lung lobes were snap-frozen and stored at -80°C for gene expression analyses. Unlavaged left lung lobes were fixed in 10% neutral buffered formalin and processed for histological analyses, as described previously (33).

### Total protein and dsDNA estimation

Total protein and dsDNA contents in the BALF were determined by Bradford assay (Bio-Rad, Hercules, CA) and spectrophotometric assay using NanoDrop 8000 (Thermo Fisher Scientific, Waltham, MA), respectively.

### Cytokine analyses

Cell-free BALF samples were assayed for various soluble mediators as previously described (34). Briefly, the BALF levels of various cytokines and chemokines were determined using Luminex XMAP-based assay (MCYTOMAG-70K), according to the manufacturer’s instructions (EMD Millipore, Billerica, MA). The list of cytokines and chemokines is included in **Supplementary Table 2**.

### Enzyme-linked immunosorbent assay

IL-13 levels were analyzed in the cell-free BALF samples using Mouse IL-13 ELISA Kit-Quantikine (Cat # M1300CB, R&D systems, Minneapolis, MN), according to the manufacturer’s instructions.

### Bacterial burden analyses

The aseptically harvested BALF was serially diluted and plated onto Columbia blood agar (CBA) plates (Hardy Diagnostics, Santa Maria, CA). The plates were incubated in anaerobic candle jars at 37°C for 48h. The CFUs were counted, and the morphological characteristics of the colonies were recorded, as previously described (32).

### Histopathological analyses

The formalin-fixed left lung lobes were embedded in paraffin and sectioned at 5µm thickness. Alcian blue-periodic acid-Schiff (AB-PAS) staining was performed to stain mucopolysaccharide materials in the airway lumen and mucins content within the airway epithelial cells. Mucus obstruction was graded using the histological semiquantitative grading strategy as previously described (32). For MCM analyses, the photographs of AB-PAS-stained large airways were captured under the 40X objective of the ECLIPSE Ci-L microscope with DS-Fi2 camera attachment (Nikon, Melville, NY). Thereafter, the analyses were performed by quantifying the number of mucous cells per millimeter (mm) of basement membrane using the Fiji software (35). All the slides were graded by a board-certified anatomic pathologist in a blinded manner.

### Immunohistochemical analyses

Formalin-fixed paraffin-embedded left lung sections were used for the immunohistochemical localization of MUC5AC, MUC5B, and E-cadherin. The sections were stained with the corresponding primary antibodies: rabbit polyclonal MUC5AC antibody (UNC 294, a kind gift by Dr. Camille Ehre, University of North Carolina, Chapel Hill, NC), rabbit polyclonal MUC5B antibody (UNC223, a kind gift by Dr. Camille Ehre, University of North Carolina, Chapel Hill, NC), and Rabbit monoclonal E-cadherin primary antibody (3195, Cell Signaling Technology, Danvers, MA) using previously published procedure (36–38). The immunostained slides were analyzed by a board-certified anatomic pathologist without prior knowledge of genotypes. The photographs were captured under the 40X or 4X objective of the ECLIPSE Ci-L microscope with DS-Fi2 camera attachment (Nikon, Melville, NY). Thereafter, captured images were processed using the Fiji software (35) to determine the percentage of stained area (MUC5B and MUC5AC).

### Gene expression analyses

Total RNA isolation from right lungs, analysis of quantity and purity of isolated total RNA, cDNA generation, and reverse transcription quantitative polymerase chain reaction (RT-qPCR) were done as described previously (39). The nucleotide sequences of primers used in RT-qPCR are included in **Supplementary Table 1**.

### BaseScope (RNA *in situ* hybridization) assay for the detection of *Egfr* mRNA

Left lungs were fixed in 10% neutral buffered formalin for 24 hrs. and embedded in paraffin. These formalin-fixed, paraffin-embedded left lung sections were used to perform RNA *in situ* localization of *Egfr* mRNA using BaseScope technology (ACD, Newark, CA). Briefly, the lung sections were baked at 60°C in a hybridization oven, deparaffinized in xylene and dehydrated in 100% ethanol followed by air-drying the slides for 5 min at 60°C. The slides were then incubated for 10 min at room temperature (RT) with 3% hydrogen peroxide (322335; ACD, Newark, CA) to quench the endogenous peroxidase activity. Target retrieval was performed using a 1X RNAscope Target Retrieval Reagent (322000; ACD, Newark, CA) at 98-102°C for 15 minutes, followed by three washes in distilled water. RNAscope Protease IV (322336; ACD, Newark, CA) was added to the lung sections and incubated at 40°C in a hybridization oven for 30 minutes followed by two washes of distilled water. Custom designed *Egfr* probe (1307511-C1; ACD, Newark, CA) equilibrated to room temperature was subsequently added to the lung sections and incubated in a hybridization oven for 2 hours at 40°C. After two washes with the wash buffer (310091, ACD, Newark, CA), the sections underwent eight amplification steps to enhance the signals. BaseScopeTM Detection Reagents v2– RED (Cat. No. 323910; ACD, Newark, CA) were used to detect the red signal. Finally, the sections were counterstained with 50% Gill’s Hematoxylin I for 2 min at RT followed by 3–5 times rinse with tap water. The slides were further washed 2–3 times in 0.02% Ammonia water, followed by 3-time wash in tap water. The slides were dehydrated at 60°C in a hybridization oven until the slides were completely dry, and coverslipped using VectaMount mounting media (H-5000; Vector Laboratories, Burlingame, CA). The stained slides were analyzed by a board-certified anatomic pathologist without prior knowledge of genotypes. The stained slides were captured under the 100X objective of the ECLIPSE Ci-L microscope with DS-Fi2 camera attachment (Nikon, Melville, NY).

### Statistical analyses

One-way analysis of variance (ANOVA) followed by Tukey’s *post hoc* test was used to determine statistically significant differences among the groups. All data were presented as mean ± standard error of the mean (SEM). Grubbs’ test was used to remove the outliers. To reduce the number of horizontal bars indicating statistically significant differences between experimental groups, we used a single bar when one group showed significant differences compared to multiple other groups (with vertical ticks). A *p*-value of less than 0.05 was considered statistically significant. All statistical analyses were performed using GraphPad Prism 10.0 (GraphPad Software Inc., La Jolla, CA).

## Results

### EGFR deletion in airway epithelial cells increases postnatal mortality in Tg+ mice

**Figure 1:**
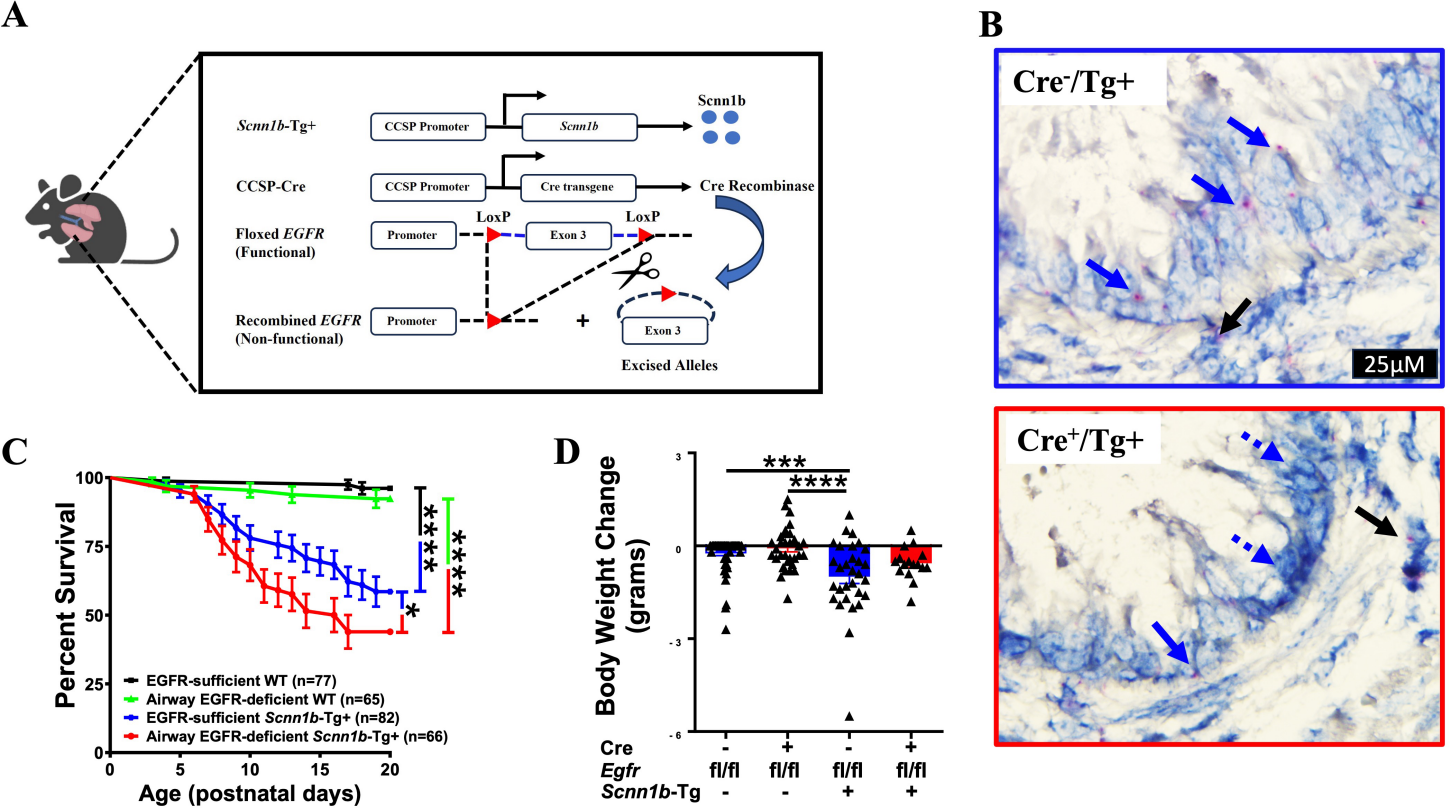
EGFR deletion in airway epithelial cells increases postnatal mortality but does not affect body weight change in *Scnn1b*-Tg+ (Tg+) mice. **(A)** Schematic diagram for the generation of airway epithelial cell-specific EGFR-deficient mice. Airway epithelial cell-specific EGFR-deficient *Scnn1b*-Tg+ (CCSP-Cre^+^/*Egfr* ^fl/fl^/Tg+) mice were generated by interbreeding club cell-specific Cre recombinase (CCSP-Cre^+^), floxed *Egfr* (*Egfr* ^fl/fl^), and *Scnn1b*-Tg+ mice. **(B)** *In situ BaseScope* hybridization for *Egfr* transcripts [red dots representing punctate staining for *Egfr* mRNA in airway epithelial cells (blue arrows) and alveolar epithelial cells (black arrows)] in EGFR-sufficient Tg+ (top panel) and airway epithelial cell-specific EGFR-deficient (bottom panel) Tg+ mice. **(C)** Survival curve for the progeny of the crosses between CCSP-Cre^+^/*Egfr* ^fl/fl^/WT and CCSP-Cre^-^/*Egfr* ^fl/fl^/Tg+ (or CCSP-Cre^+^/*Egfr^f^* ^l/fl^/Tg+ and CCSP-Cre^-^/*Egfr* ^fl/fl^/WT) mice. n= number of pups per genotype. ****p < 0.0001 (EGFR-sufficient WT (CCSP-Cre^-^/*Egfr* ^fl/fl^/WT; Cre^-^/WT) vs EGFR-sufficient *Scnn1b*-Tg+ (CCSP-Cre^-^/*Egfr* ^fl/fl^/Tg+; Cre^-^/Tg+)) and (EGFR-deficient WT (CCSP-Cre^+^/*Egfr* ^fl/fl^/WT; Cre^+^/WT) vs EGFR-deficient *Scnn1b*-Tg+ (CCSP-Cre^+^/*Egfr* ^fl/fl^/Tg+; Cre^+^/Tg+)), *p < 0.05 (Cre^-^/Tg+ vs Cre^+^/Tg+) by Gehan-Breslow-Wilcoxon test. Cre^-^/WT (black), Cre^+^/WT (green), Cre^-^/Tg+ (blue), and Cre^+^/Tg+ neonates (red). **(D)** Body weight change values represent body weight gain (positive value) or loss (negative value) as compared to gender- and age- matched Cre^-^/WT littermates. The body weights of the healthiest Cre^-^/WT male or female mice were set to 0. Individual body weight values for other littermates were generated by subtracting the body weight of Cre^-^/WT from body weight of each gender-matched littermates. Cre^-^/WT [blue open bar], Cre^+^/WT [red open bar], Cre^-^/Tg+ [solid blue bar], and Cre^+^/Tg+ [solid red bar] mice. Sample size (n=16-59/group). Error bars represent Mean ± SEM. One-way ANOVA followed by Tukey’s *post hoc* test was used for the statistical analysis. ***p < 0.001, ****p < 0.0001.

The airway epithelial cell-specific EGFR-deficient Tg+ (Cre^+^/Tg+) and control (Cre^-^/Tg+, Cre^+^/WT, and Cre^-^/WT) mice were generated (**Figure 1A**), and three-week-old juveniles (Postnatal day 21; PND21) were assessed for endpoints including postnatal survival, RT-qPCR for genes relevant to mucoinflammatory responses, mucus obstruction, MCM, immune cell recruitment, cytokine levels, and bacterial load. The deletion of EGFR in the airway epithelial cells was confirmed by BaseScope assay (**Figure 1B**).

The germline deletion of EGFR in mice results in mortality between midgestation and PND 20, depending on the genetic background of the mice (14). Therefore, to determine whether the airway-epithelial cell-specific EGFR deficiency contributes to the embryonic mortality, we analyzed the possibility of embryonic mortality by calculating the mendelian ratio of the expected genotypes of neonates, i.e., Cre^-^/WT (n=80), Cre^+^/WT (n=72), Cre^-^/Tg+ (n=83), and Cre^+^/Tg+ (n=66). The mendelian ratio for the Cre^-^/WT, Cre^+^/WT, Cre^-^/Tg+, and Cre^+^/Tg+ progeny was 1.06:0.96:1.10:0.88, respectively. The calculated χ2 value of 0.498 suggested that the observed mendelian ratio was not significantly deviated from the expected mendelian ratio of 1:1:1:1. These data suggest that airway epithelial cell-specific deletion of EGFR doesn’t compromise embryonic viability.

The Tg+ mice exhibit significant postnatal mortality within the first 3 weeks of life (11, 26, 27). To determine the effect of airway epithelial cell-specific EGFR deletion on postnatal survivability of the Tg+ mice, we observed pups until PND20. The survivability was comparable between Cre^-^/WT and Cre^+^/WT pups (**Figure 1C**), suggesting that airway epithelial cell-specific EGFR deletion does not compromise postnatal survival in WT pups. Consistent with the previous studies (11, 26, 27, 39), Cre^-^/Tg+ exhibited ~41.5% mortality between PND0-PND20, with most of the deaths observed between PND7-PND17 (**Figure 1C**). As compared with the Cre^-^/Tg+ group, the Cre^+^/Tg+ mice showed significantly higher mortality rate of ~56.1% (**Figure 1C**). However, similar to the Cre^-^/Tg+ group, most of the deaths in Cre^+^/Tg+ mice occurred between PND7-PND17. We also investigated the effect of airway epithelial cell-specific deletion of EGFR on postnatal distress in mice by recording their body weights at PND21. Although the Tg+ pups of both genotypes displayed lower body weight at PND21, the airway epithelial cell-specific deletion of EGFR did not alter the postnatal body weight in WT and Tg+ juveniles (**Figure 1D**). These data suggest that the EGFR deletion promotes mucus obstruction, a pathogenic pathway that contributes to the neonatal mortality in Tg+ mice (11, 26, 27).

### EGFR deletion in airway epithelial cells increases expression of type 2 inflammation-associated gene signatures and mucus obstruction in Tg+ juveniles

The Tg+ mice predominantly exhibit type 2 inflammation characterized by increased levels of Th2 cytokines, i.e., IL-4 and IL-13, and elevated type 2 inflammation-associated gene signatures (27, 30, 32, 39). Indeed, type 2 inflammation-associated mucus obstruction is known to be the primary cause of postnatal mortality in Tg+ mice (11, 26, 27). Here, we hypothesized that the exaggerated type 2 inflammation in the Cre^+^/Tg+ mice contributes to excessive mucus obstruction, which results in their increased mortality.

**Figure 2:**
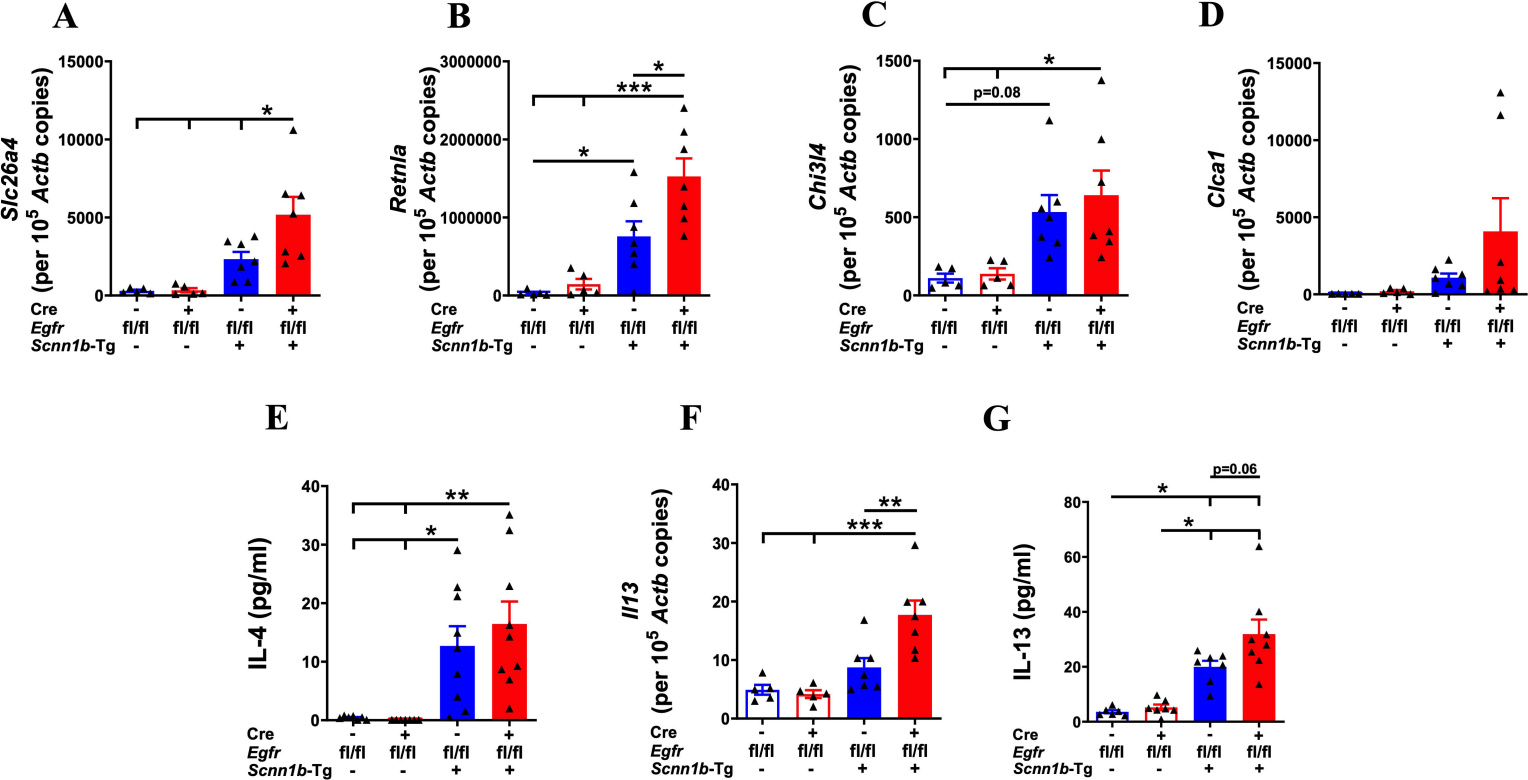
EGFR deletion in airway epithelial cells increases the expression of markers associated with type 2 inflammation in Tg+ juveniles. Absolute quantification of *Slc26a4* mRNA **(A)**, *Retnla* mRNA **(B)**, *Chi3l4* mRNA **(C)**, and *Clca1* mRNA **(D)** in the lungs from WT mice (with Cre^-^ or Cre^+^ status) and Tg+ mice (with Cre^-^ or Cre^+^ status) (n=5-7/group). BALF cytokine levels (in pg/ml; picograms per milliliter) of IL-4 (n=7-9/group) **(E)** and absolute quantification of *Il13* mRNA **(F)** in the lungs from WT mice (with Cre^-^ or Cre^+^ status) and Tg+ mice (with Cre^-^ or Cre^+^ status). Sample size (n=5-7/group). **(G)** BALF cytokine levels (in pg/ml; picograms per milliliter) of IL-13 (n=6-8/group) in the lungs from WT mice (with Cre^-^ or Cre^+^ status) and Tg+ mice (with Cre^-^ or Cre^+^ status). Error bars represent Mean ± SEM. One-way ANOVA followed by Tukey’s *post hoc* test was used for the statistical analysis. *p < 0.05, **p < 0.01, ***p < 0.001. To minimize the number of horizontal lines in various panels (e.g., **A**), single horizontal line with small vertical ticks were used to indicate statistically significant differences. The positioning of small vertical ticks indicates the group that is significantly different from the reference group (with no vertical line). In **(A)**, single horizontal significance line with three vertical ticks suggests significant difference for three comparisons, i.e., Cre^+^/Tg+ vs Cre^-^/Tg+, Cre^+^/Tg+ vs Cre^+^/WT, and Cre^+^/Tg+ vs Cre^-^/WT.

First, we analyzed the expression levels of type 2 inflammation-associated gene signatures, including *Slc26a4*, *Retnla, Chi3l4*, and *Clca1*. The mRNA levels for *Slc26a4*, *Retnla, Chi3l4*, and *Clca1* were comparable in Cre^-^/WT and Cre^+^/WT mice (**Figures 2A–D**), which suggest that the EGFR deletion in the healthy airways does not induce type 2 inflammation. As reported previously (30, 32), the *Slc26a4* mRNA levels trended higher in Cre^-^/Tg+ mice as compared with Cre^-^/WT and Cre^+^/WT mice (**Figure 2A**). The *Slc26a4* mRNA levels were significantly higher in Cre^+^/Tg+ mice as compared with all the other three experimental groups (**Figure 2A**). The mRNA levels of *Retnla* and *Chi3l4* were higher in Cre^-^/Tg+ versus Cre^-^/WT mice (**Figures 2B, C**). As compared with the Cre^-^/Tg+ mice, the mRNA levels of *Retnla, Chi3l4*, and *Clca1* were higher in Cre^+^/Tg+ mice, with only *Retnla* showing a significant increase (**Figures 2B–D**). Because IL4Rα, a common receptor subunit for IL-4 and IL-13, is known to cause mucous cell metaplasia in Tg+ mice (11), we assessed the levels of IL-4 and IL-13. The IL-4 protein levels were comparable in BALF from Cre^-^/WT and Cre^+^/WT mice (**Figure 2E**). As reported previously (32, 40), Cre^-^/Tg+ mice had elevated IL-4 levels as compared with their WT counterparts (**Figure 2E**). As compared with the Cre^-^/Tg+ mice, the Cre^+^/Tg+ mice had insignificantly elevated (p =0.77) IL-4 levels (**Figure 2E**). The *Il13* mRNA levels in the lung homogenates were comparable among Cre^-^/WT and Cre^+^/WT mice and significantly higher in the Cre^+^/Tg+ group versus the other three experimental groups (**Figure 2F**). Consistent with the mRNA levels, the IL-13 protein levels showed higher trend in Cre^+^/Tg+ mice compared to Cre^-^/Tg+ (p =0.06) (**Figure 2G**).

**Figure 3:**
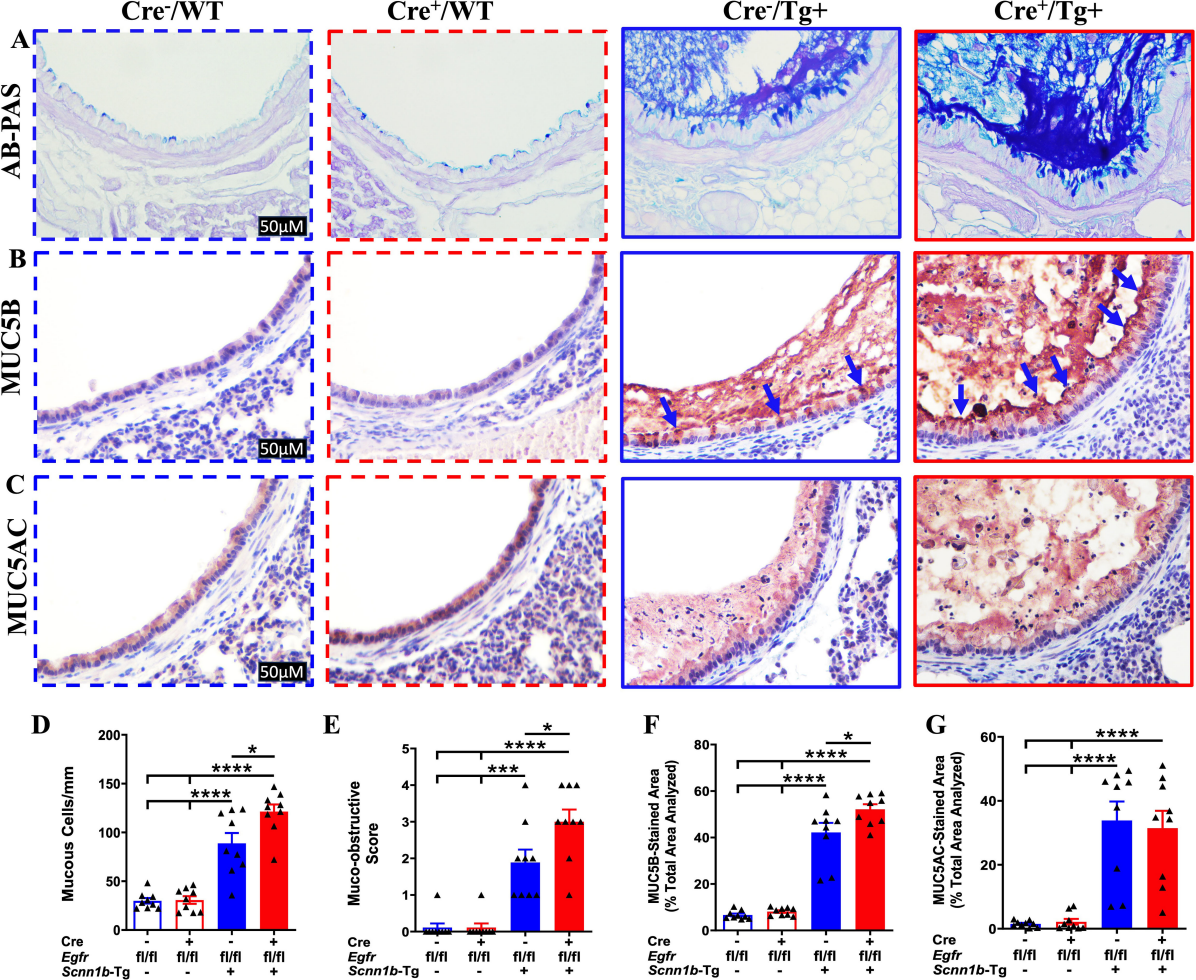
EGFR deletion in airway epithelial cells promotes mucus obstruction in Tg+ juveniles. Representative photomicrographs of AB-PAS-stained **(A)**, MUC5B-immunostained **(B)**, and MUC5AC-immunostained **(C)** left lung lobe sections from Cre^-^/WT, Cre^+^/WT, Cre^-^/Tg+, and Cre^+^/Tg+ mice. All photomicrographs for each stain across all four groups were taken at the same magnification. (Cre^-^/WT [blue dotted border], Cre^+^/WT [red dotted border], Cre^-^/Tg+ [blue solid border], and Cre^+^/Tg+ [red solid border] mice). Blue arrows indicate MUC5B+ airway epithelial cells. **(D)** Semiquantitative histological scoring for airway mucus obstruction from AB-PAS-stained left lung sections from Cre^-^/WT, Cre^+^/WT, Cre^-^/Tg+, and Cre^+^/Tg+ mice. Sample size (n=9/group). **(E)** Number of mucous cells per millimeter of basement membrane from AB-PAS-stained left lung sections from Cre^-^/WT, Cre^+^/WT, Cre^-^/Tg+, and Cre^+^/Tg+ mice. Sample size (n=9/group)**. (F)** Percent MUC5B-stained area in the first-generation airway section calculated using Fiji software. Sample size (n=9/group). **(G)** Percent MUC5AC-stained area in the first-generation airway section was calculated using Fiji software. Sample size (n=9/group). Different groups are shown as: Cre^-^/WT (blue open bar), Cre^+^/WT (red open bar), Cre^-^/Tg+ (blue solid bar), and Cre^+^/Tg+ (red solid bar). Error bars represent Mean ± SEM. One-way ANOVA followed by Tukey’s *post hoc* test was used for the statistical analysis. *p < 0.05, ***p < 0.001, ****p < 0.0001. To minimize the number of horizontal lines in various panels (e.g., **D**), single horizontal line with small vertical ticks were used to indicate statistically significant differences. The positioning of small vertical ticks indicates the group that is significantly different from the reference group (with no vertical line). In **(D)**, single horizontal significance line with three vertical ticks suggests significant difference for two comparisons, i.e., Cre^-^/Tg+ vs Cre^+^/WT, and Cre^-^/Tg+ vs Cre^-^/WT.

Airway mucous cell metaplasia (MCM) and mucus obstruction are consistent features of lung disease in Tg+ mice, where two gel-forming mucins, i.e., MUC5B and MUC5AC, primarily contribute to airway mucus obstruction (27). To investigate the effect of airway epithelial cell-specific EGFR deletion on MCM and mucus obstruction, we performed AB-PAS, MUC5B, and MUC5AC staining on the lung sections. While only a very small proportion of Cre^-^/WT and Cre^+^/WT mice showed minimal airway mucus obstruction, Cre^-^/Tg+ mice had a marked increase in the proportion of mucous cells (AB-PAS+, MUC5B+, and MUC5AC+) and the degree of airway mucus obstruction (**Figures 3A–G**, **Supplementary Figure 1**). The extent of MCM, as indicated by the proportion of AB-PAS+ and MUC5B+ airway epithelial cells, and the degree of airway mucus obstruction, as indicated by AB-PAS- and MUC5B-stained airway luminal contents, were significantly higher in Cre^+^/Tg+ mice compared with Cre^-^/Tg+ mice (**Figures 3A, B, D–F**). The proportion of MUC5AC+ airway epithelial cells was comparable between Cre^+^/Tg+ mice versus Cre^-^/Tg+ mice (**Figures 3C, G**). These data suggest that airway epithelial cell-specific EGFR deficiency results in increased MUC5B production and mucus obstruction in Tg+ juveniles.

### EGFR deletion in airway epithelial cells worsens inflammatory features in Tg+ juveniles

**Figure 4:**
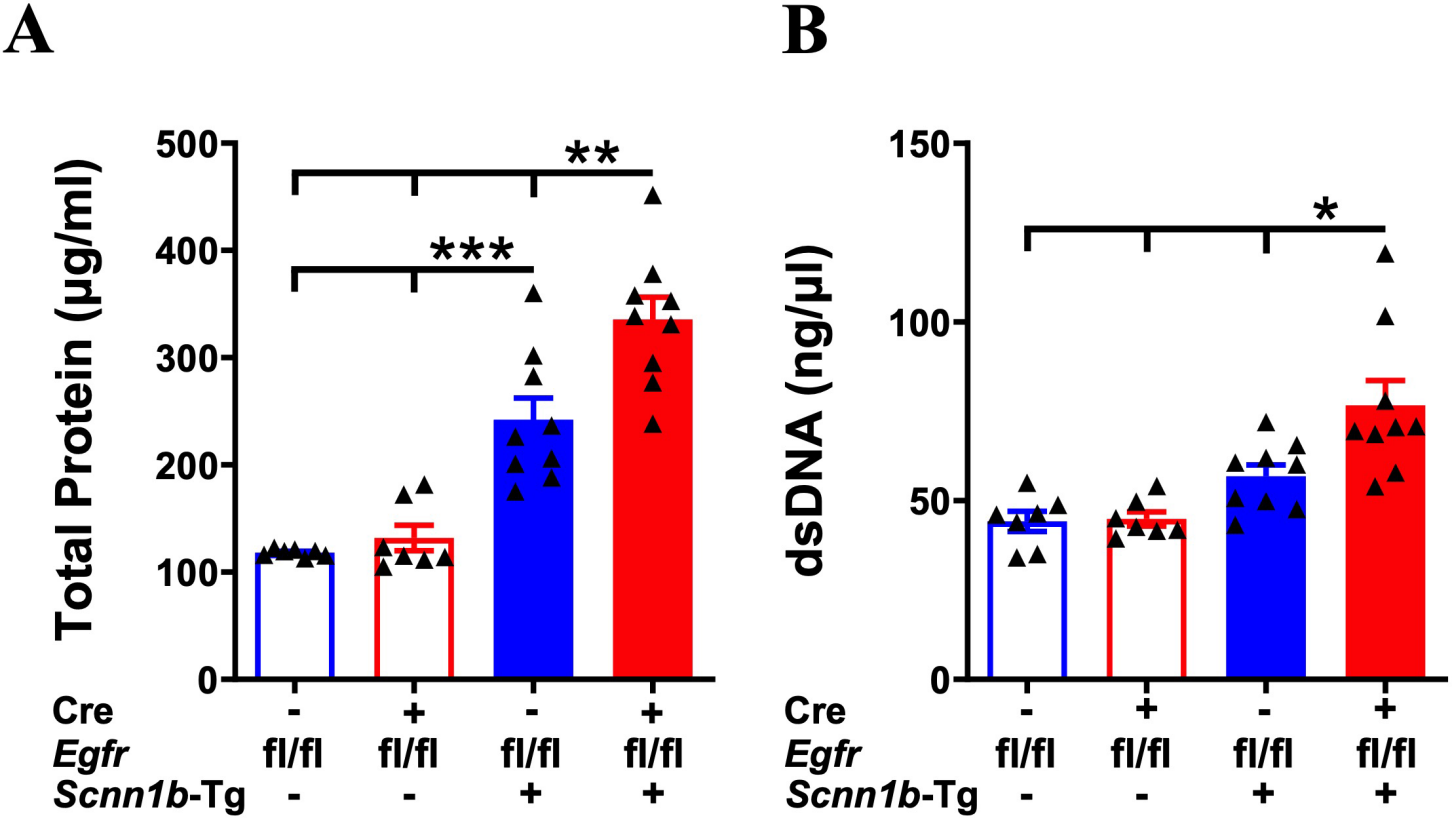
Airway epithelial cell-specific EGFR-deficient Tg+ mice exhibit elevated BALF protein and dsDNA contents. The total protein contents (µg/ml) **(A)** and dsDNA contents (ng/µl) **(B)** in cell-free BALF from WT mice (with Cre^-^ or Cre^+^ status) and Tg+ mice (with Cre^-^ or Cre^+^ status). Sample size (n=7-9/group). Error bars represent Mean ± SEM. One-way ANOVA followed by Tukey’s *post hoc* test was used for the statistical analysis. *p < 0.05, **p < 0.01, ***p < 0.001. To minimize the number of horizontal lines in various panels (e.g., **A**), single horizontal line with small vertical ticks were used to indicate statistically significant differences. The positioning of small vertical ticks indicates the group that is significantly different from the reference group (with no vertical line). In **(A)**, single horizontal significance line with three vertical ticks suggests significant difference for two comparisons, i.e., Cre^-^/Tg+ vs Cre^+^/WT, and Cre^-^/Tg+ vs Cre^-^/WT.

Increased total protein and dsDNA contents in the BALF are indicative of lung inflammation (32, 41, 42). Therefore, to determine the effect of airway epithelial cell-specific EGFR deletion on lung inflammation, we assessed total protein and dsDNA contents in the BALF. BALF total protein and dsDNA contents were comparable among Cre^-^/WT and Cre^+^/WT mice (**Figures 4A, B**). Cre^-^/Tg+ mice, on the other hand, had significantly elevated BALF total protein and an increasing trend of dsDNA contents, as compared with Cre^-^/WT and Cre^+^/WT mice (**Figures 4A, B**). Deletion of EGFR in the airway epithelial cells significantly increased the BALF total protein and dsDNA contents in Cre^+^/Tg+ mice compared with all other experimental groups (**Figures 4A, B**), suggesting that airway epithelial cell-specific deletion of EGFR contributes to exaggerated lung inflammation in Tg+ juveniles.

To determine whether the increase in the BALF protein levels in Cre^+^/Tg+ mice is caused by epithelial barrier dysfunction, we assessed the mRNA levels of key apical junction complex proteins that are critical for epithelial barrier function. The mRNA levels of genes encoding adherens junction (AJ) proteins, i.e., E-Cadherin (*Cdh1*), were significantly reduced in Cre^+^/Tg+ mice as compared with Cre^-^/Tg+ mice (**Supplementary Figure 2A**). The mRNA levels of genes encoding tight junction (TJ) proteins, including Claudin 5 (*Cldn5*), Occludin (*Ocln*), and ZO1 (*Tjp1*), adherens junction (AJ) proteins, i.e., Beta-catenin (*Ctnnb1*), and gap junction protein, i.e., Connexin 43 (*Cx43*) trended lower in Cre^+^/Tg+ mice as compared with Cre^-^/Tg+ mice (**Supplementary Figures 2B–F**). Consistent with the reduced *Cdh1* mRNA levels, the immunohistochemical staining of E -Cadherin showed reduced staining intensity in Cre^+^/Tg+ mice as compared with Cre^-^/Tg+ mice (**Supplementary Figure 2G**).

**Figure 5:**
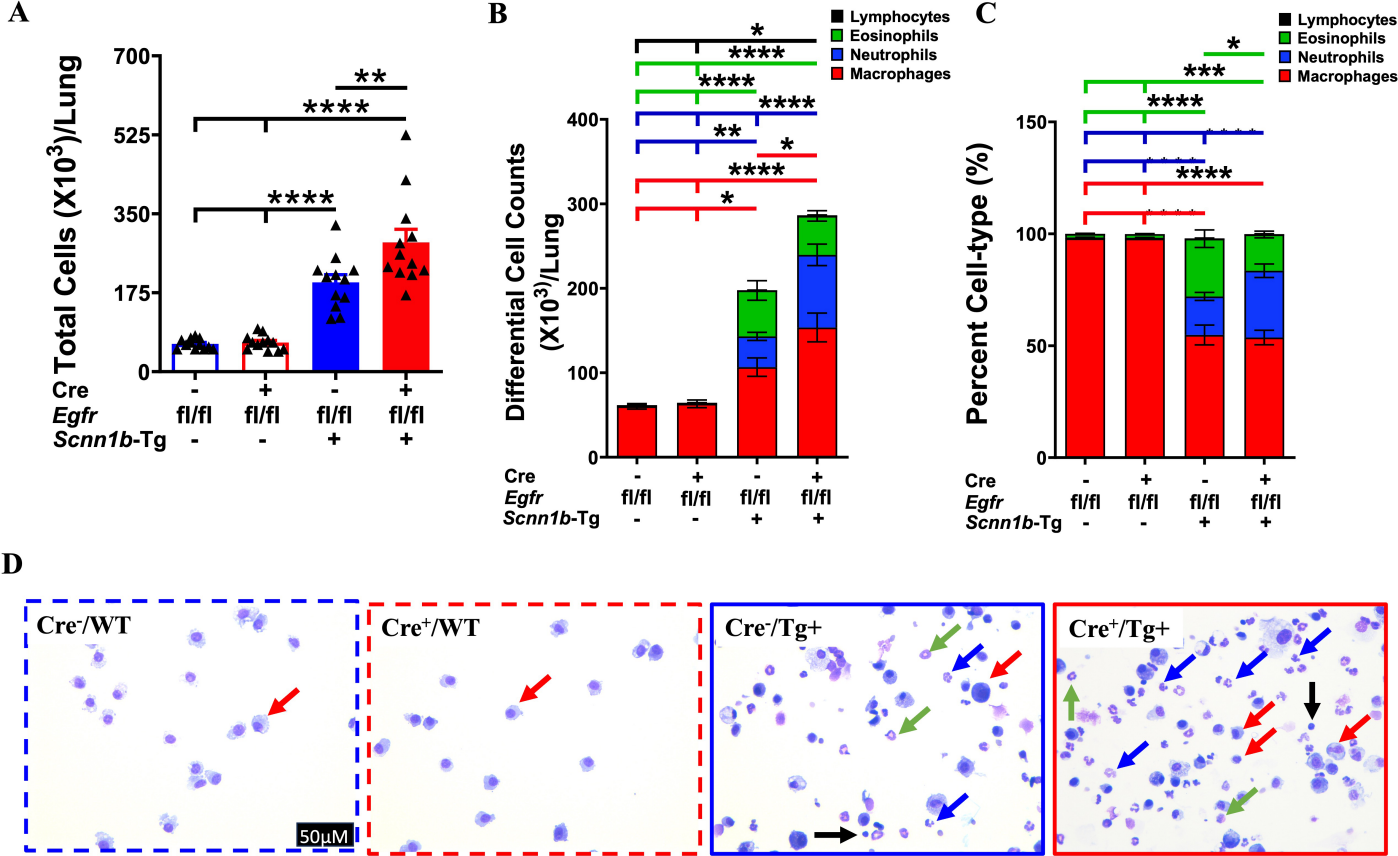
EGFR deletion in airway epithelial cells modulates immune cell recruitment in airspaces of Tg+ mice. Total cell counts **(A)** are shown for Cre^-^/WT [blue open bar], Cre^+^/WT [red open bar], Cre^-^/Tg+ [solid blue bar], and Cre^+^/Tg+ [solid red bar] mice (n=12/group). Differential cell counts **(B)** and the relative percentages **(C)** are presented as a stacked bar graph (macrophages [red bar], neutrophils [blue bar], eosinophils [green bar], and lymphocytes [black bar]) in the harvested BALF from Cre^-^/WT, Cre^+^/WT, Cre^-^/Tg+, and Cre^+^/Tg+ mice. Sample size (n=11-12/group). Error bars represent Mean ± SEM. One-way ANOVA followed by Tukey’s *post hoc* test was used for the statistical analysis. *p < 0.05, **p < 0.01, ***p < 0.001, ****p < 0.0001. **(D)** Representative photomicrographs of Wright-Giemsa-stained BALF cytospins from Cre^-^/WT, Cre^+^/WT, Cre^-^/Tg+, and Cre^+^/Tg+ mice. Macrophages (red arrows), neutrophils (blue arrows), eosinophils (green arrows), and lymphocytes (black arrows). All photomicrographs in panel D were taken at the same magnification. To minimize the number of horizontal lines in various panels (e.g., **A**), single horizontal line with small vertical ticks were used to indicate statistically significant differences. The positioning of small vertical ticks indicates the group that is significantly different from the reference group (with no vertical line). In **(A)** single horizontal significance line with three vertical ticks suggests significant difference for two comparisons, i.e., Cre^-^/Tg+ vs Cre^+^/WT, and Cre^-^/Tg+ vs Cre^-^/WT.

Next, we determined the effect of airway epithelial cell-specific EGFR deletion on immune cell recruitment into the airspaces by performing immune cell analyses in the BALF from all four experimental groups. Cre^-^/WT and Cre^+^/WT mice had comparable total immune cells in the BALF (**Figures 5A, D**). The proportion of four types of immune cells, i.e., macrophages, neutrophils, eosinophils, and lymphocytes, were also comparable among Cre^-^/WT and Cre^+^/WT mice (**Figures 5B–D**). These data suggest that the airway epithelial cell-specific deletion of EGFR doesn’t alter immune cell composition in the airspaces of WT mice. Consistent with previous reports (26, 27, 32, 40), as compared with the Cre^-^/WT and Cre^+^/WT mice, the total cell counts were significantly increased in Cre^-^/Tg+ mice, which was mainly attributable to increase in macrophages, neutrophils, eosinophils, and lymphocytes (**Figures 5A–D**). The total cell counts were significantly increased in Cre^+^/Tg+ as compared with Cre^-^/Tg+ mice (**Figures 5A–D**), which was attributable to the increased numbers of macrophages and neutrophils (**Figures 5B–D**). These data suggest that airway epithelial cell-specific deficiency of EGFR enhances neutrophil and macrophage recruitment into the airspaces of Tg+ mice.

**Figure 6:**
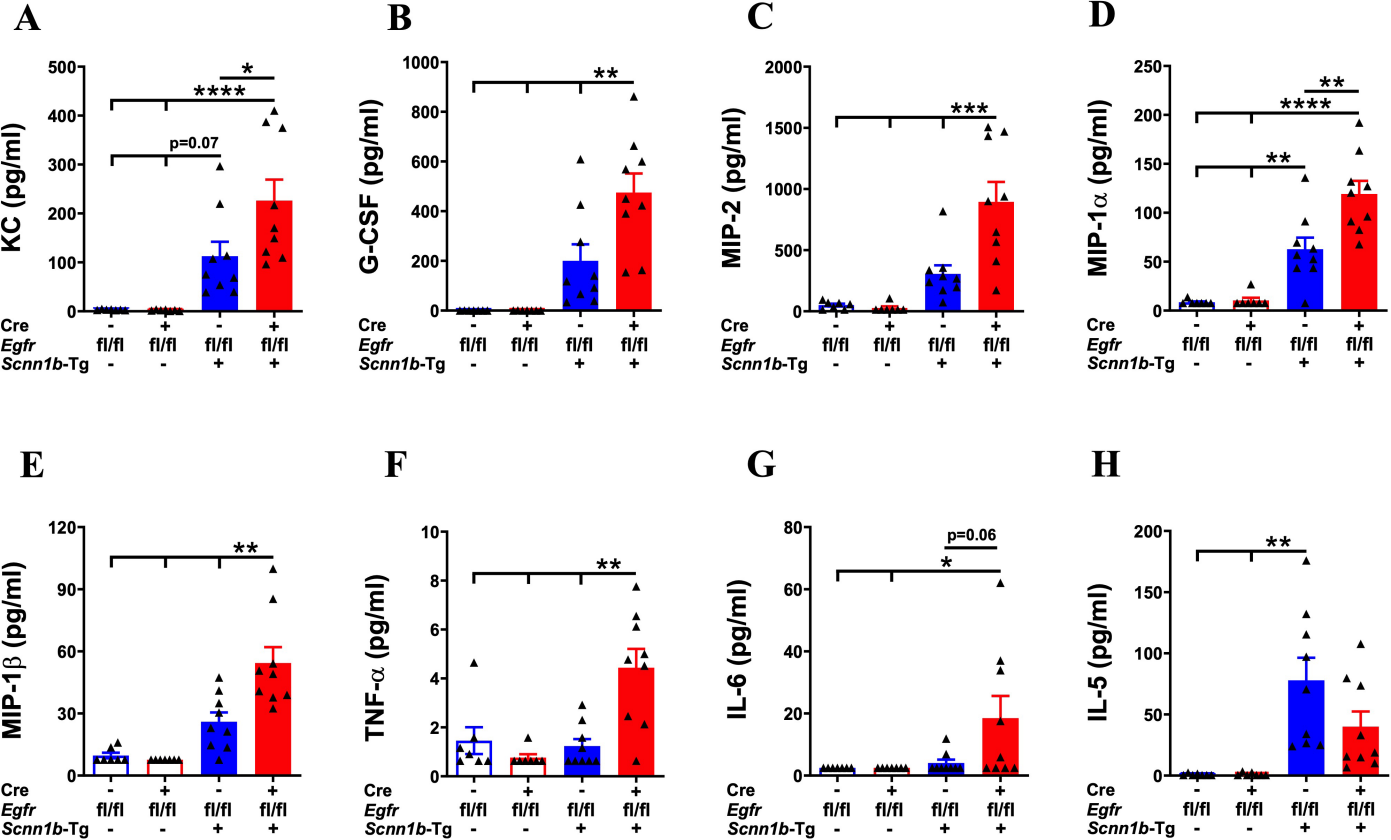
EGFR deletion in airway epithelial cells alters the levels of inflammatory mediators in the airspaces of Tg+ mice. Cell-free BALF cytokine levels (pg/ml; picograms per milliliter) of KC **(A)**, G-CSF **(B)**, MIP-2 **(C)**, MIP-1α **(D)**, MIP-1β **(E)**, TNF-α **(F)**, IL-6 **(G)**, and IL-5 **(H)** in WT mice (with Cre^-^ or Cre^+^ status) and Tg+ mice (with Cre^-^ or Cre^+^ status). Sample size (n=7-9/group). Error bars represent Mean ± SEM. One-way ANOVA followed by Tukey’s *post hoc* test was used for the statistical analysis. *p < 0.05, **p < 0.01, ***p < 0.001, ****p < 0.0001. To minimize the number of horizontal lines in various panels (e.g., **B**), single horizontal line with small vertical ticks were used to indicate statistically significant differences. The positioning of small vertical ticks indicates the group that is significantly different from the reference group (with no vertical line). In **(B)** single horizontal significance line with three vertical ticks suggests significant difference for three comparisons, i.e., Cre^+^/Tg+ vs Cre^-^/Tg+, Cre^+^/Tg+ vs Cre^+^/WT, and Cre^+^/Tg+ vs Cre^-^/WT.

Next, we assessed the effect of airway epithelial cell-specific EGFR deletion on inflammatory mediators released into the lung airspaces by determining BALF cytokines and chemokines levels. The levels of primary neutrophil-specific chemokines, including KC/CXCL1, G-CSF, and MIP-2/CXCL2, were comparable among Cre^-^/WT and Cre^+^/WT mice (**Figures 6A–C**). Consistent with the increased neutrophil counts in Cre^-^/Tg+ mice, KC/CXCL1, G-CSF, and MIP-2/CXCL2 levels trended higher in Cre^-^/Tg+ compared with Cre^-^/WT and Cre^+^/WT mice (**Figures 6A–C**). Mirroring the additional increase in neutrophil counts in Cre^+^/Tg+ mice compared with Cre^-^/Tg+ mice, KC/CXCL1, G-CSF, and MIP-2/CXCL2 levels were significantly increased in Cre^+^/Tg+ mice compared with Cre^-^/Tg+ mice (**Figures 6A–C**). Consistent with the increased macrophage counts, the levels of MIP-1α/CCL3 and MIP-1β/CCL4, the chemokines reported to drive macrophage/monocyte recruitment (43–45), were significantly increased in Cre^+^/Tg+ mice compared with all other three experimental groups (**Figures 6D, E**). Other inflammatory mediators, such as TNF-α, showed significantly higher expression levels in Cre^+^/Tg+ mice compared with all other experimental groups (**Figure 6F**), and IL-6 showed significantly higher expression levels in Cre^+^/Tg+ mice compared with WT groups and a higher trend in Cre^+^/Tg+ mice than Cre^-^/Tg+ mice (**Figure 6G**). We also assessed the levels of IL-5, a type 2 cytokine that plays a key role in the proliferation, maturation, and differentiation of eosinophils (46, 47). IL-5 levels were significantly higher in Cre^-^/Tg+ mice and trended higher in Cre^+^/Tg+ mice versus their WT counterparts (**Figure 6H**). Consistent with the comparable eosinophil counts, IL-5 levels did not differ significantly between Cre^-^/Tg+ and Cre^+^/Tg+ mice (**Figure 6H**). These data suggest that the airway epithelial cell-specific deletion of EGFR promotes a pro-inflammatory microenvironment in Tg+ airways that dictates the recruitment of inflammatory immune cells into the airspaces.

### EGFR deletion in airway epithelial cells compromises bacterial clearance in airspaces of Tg+ mice

**Figure 7:**
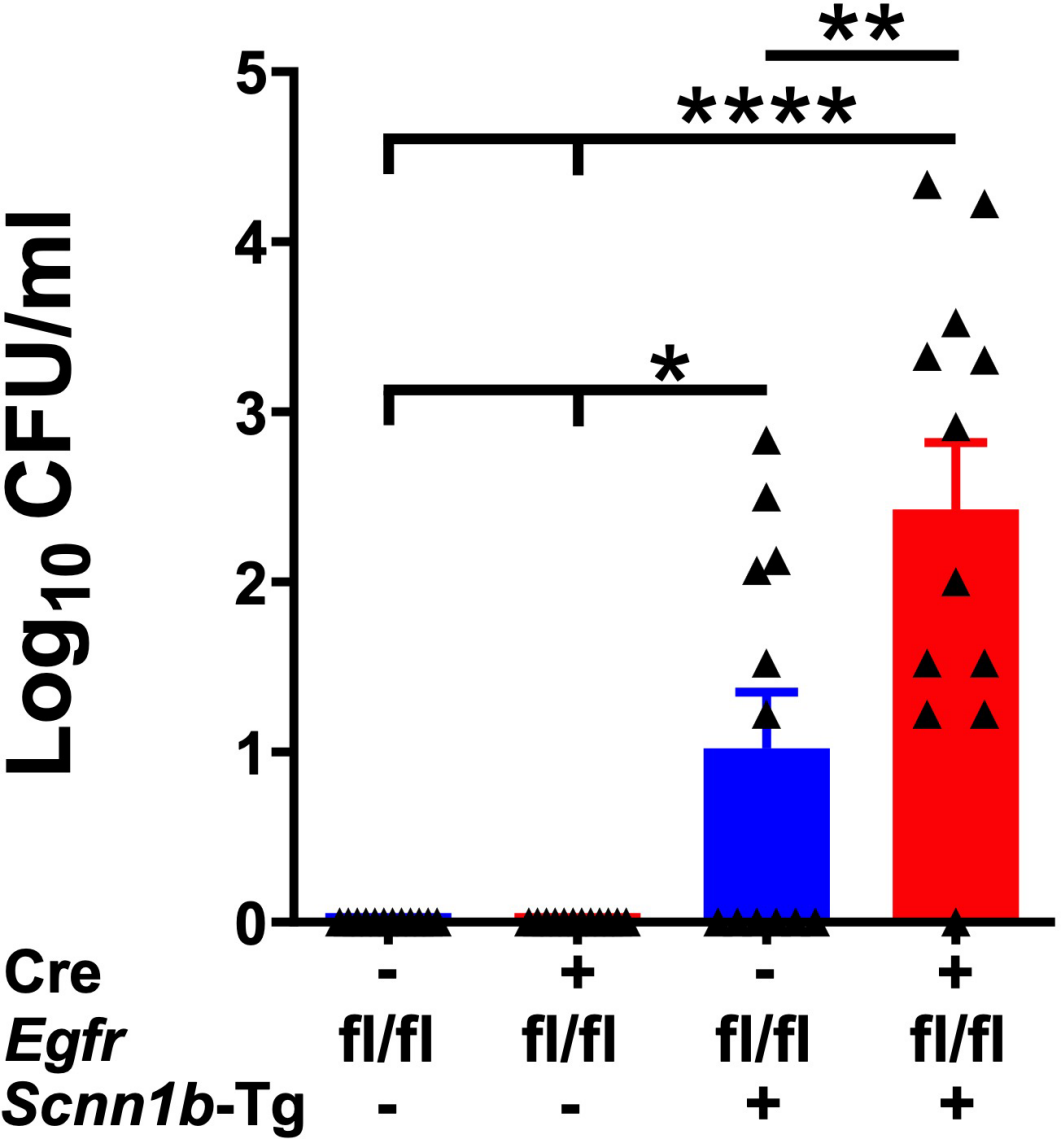
Airway epithelial cell-specific deletion of EGFR compromises bacterial clearance in Tg+ mice. Colony Forming Units (CFU) were counted in BALF from Cre^-^/WT [blue open bar], Cre^+^/WT [red open bar], Cre^-^/Tg+ [solid blue bar], and Cre^+^/Tg+ [solid red bar] mice. The CFU values were log10-transformed. Sample size (n=12/group). Error bars represent Mean ± SEM. One-way ANOVA followed by Tukey’s *post hoc* test was used for the statistical analysis. *p < 0.05, **p < 0.01, ****p < 0.0001. To minimize the number of horizontal lines in various panels, single horizontal line with small vertical ticks were used to indicate statistically significant differences. The positioning of small vertical ticks indicates the group that is significantly different from the reference group (with no vertical line). In **Figure 7**, single horizontal significance line with three vertical ticks suggests significant difference for two comparisons, i.e., Cre^-^/Tg+ vs Cre^+^/WT, and Cre^-^/Tg+ vs Cre^-^/WT.

Spontaneous bacterial infections due to a defect in the mucociliary clearance resulting from mucostasis are a consistent feature of Tg+ lung disease (29, 31, 32, 39, 40, 48). These bacterial infections are, however, reported to be cleared in the early adulthood (29). To determine the effect of airway epithelial cell-specific EGFR deletion on the clearance of spontaneous bacterial infections, we estimated airspace bacterial burden by determining the colony-forming unit (CFU) counts in BALF at PND21. The BALF collected from Cre^-^/WT and Cre^+^/WT mice were devoid of bacterial colonies (**Figure 7**). Approximately 50% (6 out of 12) of Cre^-^/Tg+ mice still had lower bacterial counts (mean CFU= ~108/ml) (**Figure 7**). In contrast, 91.6% (11 out of 12 mice) of Cre^+^/Tg+ mice showed bacterial burden (mean CFU= ~3907/ml), which was significantly higher as compared with Cre^-^/Tg+ mice (**Figure 7**). These data suggest that the airway epithelial cell-specific EGFR deletion delays bacterial clearance in Tg+ mice.

## Discussion

The EGFR signaling is implicated in the pathogenesis of airway hypersecretory and mucoinflammatory diseases such as asthma and COPD (12). The expression of EGFR and its ligands, including amphiregulin and heparin-binding EGF (HB-EGF), is increased in airway epithelial cells in asthmatics compared with non-asthmatic patients (49). EGFR signaling is also critical for inducing mucous cell metaplasia (MCM) in animal models and for mucins expression in human airway epithelial cells (6, 21–24). The levels of gel-forming mucins, i.e., MUC5AC and MUC5B, and the expression of EGFR and its ligands, i.e., TGF-α and amphiregulin, are elevated in the CF patients (25, 50). However, the pathogenic role of EGFR signaling in mucoinflammatory lung diseases remains unknown.

In this study, we investigated the role of airway epithelial cell-specific EGFR signaling in the pathogenesis of mucoinflammatory lung disease in *Scnn1b*-transgenic (Tg+) mice, a mouse model of human CF-like lung disease. More specifically, we attempted to answer the following questions: 1) Does airway epithelial cell-specific deletion of EGFR affect the respiratory tract homeostasis in WT mice? 2) Does airway epithelial cell-specific deletion of EGFR affect the embryonic and postnatal survivability in Tg+ mice? 3) Does airway epithelial cell-specific deletion of EGFR affect the MCM, mucins production, and mucus obstruction in Tg+ mice? 4) Does airway epithelial cell-specific deletion of EGFR affect the inflammatory outcomes in Tg+ mice? 5) Does airway epithelial cell-specific deletion of EGFR affect bacterial clearance in Tg+ mice? To answer these questions, we examined the effect of airway epithelial cell-specific EGFR deficiency on the manifestation of mucoinflammatory outcomes in Tg+ mice.

First, we assessed the effect of airway epithelial cell-specific EGFR deficiency on the composition of mucous cells, a cell type specialized in the synthesis and secretion of mucins (51), in WT mice. Consistent with previous studies (11, 26, 27, 32), the mucous cells were minimal and comparable in EGFR-sufficient WT (Cre^-^/WT) and airway epithelial cell-specific EGFR-deficient WT (Cre^+^/WT) mice. These data suggest that airway epithelial cell-specific EGFR deficiency does not alter the epithelial cell composition in the WT mice. Next, we assessed the effect of airway epithelial cell-specific EGFR deficiency on the numbers and composition of immune cells in the airspaces of WT mice. The Cre^-^/WT and Cre^+^/WT mice had a comparable total number of immune cells dominated by macrophages, suggesting that airway epithelial cell-specific EGFR deficiency does not alter the immune cell composition in WT mice. Additionally, the BALF levels of total protein, dsDNA, and inflammatory mediators were also comparable between Cre^-^/WT and Cre^+^/WT mice. These data suggest that airway epithelial cell-specific deletion of EGFR does not affect the respiratory tract homeostasis in WT mice.

Next, we examined the effect of airway epithelial cell-specific EGFR deficiency on the embryonic and postnatal survivability. Although the germ-line deletion of EGFR results in embryonic lethality (14), as indicated by the mendelian ratio of expected progeny obtained, the airway epithelial cell-specific EGFR deficiency did not result in any embryonic lethality. However, the airway epithelial cell-specific EGFR deficiency compromised the postnatal survival in Tg+ mice. Earlier studies have shown that the postnatal mortality in Tg+ mice is mainly attributed to sudden respiratory collapse due to airway mucus obstruction (11, 26, 27). Therefore, we next compared the severity of mucus obstruction between EGFR-sufficient Tg+ (Cre^-^/Tg+) and airway epithelial cell-specific EGFR-deficient Tg+ (Cre^+^/Tg+) mice. We found that airway epithelial cell-specific EGFR deficiency results in increased mucus obstruction in Tg+ mice, which was likely a factor in their increased postnatal mortality.

The static mucus in the Tg+ mice primarily consists of mixed mucopolysaccharide material that stains positive for two major gel-forming mucins, i.e., MUC5B and MUC5AC (31, 32, 40). The absence of MUC5B, not MUC5AC, significantly reduces the extent of mucus plugging or airway mucus obstruction in Tg+ mice, suggesting the unique contribution of MUC5B to the mucoinflammatory phenotype of these mice (52). Consistent with this report, the airway epithelial cell-specific deletion of EGFR significantly increased MUC5B levels without altering the MUC5AC levels. Our previous reports also found a strong association between MUC5B expression and mucus obstruction (39, 40). These findings suggest that increased MUC5B levels in Cre^+^/Tg+ mice might have contributed to the exaggerated mucus obstruction and postnatal mortality.

Earlier studies have shown that Tg+ mice exhibit mucus obstruction along with MCM, suggesting that MCM, in part, may contribute to mucus obstruction (26, 27, 32, 48). The mucous cells, indicative of MCM, stain positive for mucopolysaccharide material (MUC5B and MUC5AC) in the Tg+ mice (31, 32, 40). Pharmacological inhibition of EGFR signaling, using drugs such as AG-1478, gefitinib, or BIBX1522, has been demonstrated to reduce MCM in both acute and chronic asthma models (6, 53–55), suggesting a contributory role of EGFR in MCM. Consequently, we compared MCM between Cre^-^/Tg+ and Cre^+^/Tg+ mice. In contradiction to the pharmacological EGFR inhibition reports in experimental asthma models (6, 53–55), the airway epithelial cell-specific EGFR deficiency resulted in an enhanced MCM in Tg+ mice, a trend consistent with the increased mucus obstruction in these mice. These opposing effects observed between cell-specific genetic deletion and broad-spectrum pharmacological inhibition indicate that EGFR pathway in non-airway epithelial cells may also contribute to the manifestation of MCM.

IL-4 and IL-13 via IL4Rα signaling have been known to drive MCM in 10-day-old Tg+ mice (11) and mice models of allergic asthma (10, 56). In our study, the *Il13* mRNA levels were significantly upregulated in Cre^+^/Tg+ versus Cre^-^/Tg+ mice. IL-13 and IL-4 levels also showed a higher trend in Cre^+^/Tg+ versus Cre^-^/Tg+ mice. The mRNA levels of *Slc26a4* and *Retnla*, markers of type 2 inflammation, were also significantly upregulated in Cre^+^/Tg+ versus Cre^-^/Tg+ mice. These data suggest that increased IL-13 expression might have contributed in part to the increased MCM, type 2 inflammatory markers, MUC5B expression, and mucus obstruction in mice with airway epithelial cell-specific EGFR deficiency.

Airway inflammation, indicated by increased immune cell recruitment and elevated proinflammatory mediators, is a consistent feature of CF and Tg+ airways (26, 27, 31, 32, 39, 40, 48, 57–60). In the current study, the airway epithelial cell-specific EGFR deficiency resulted in increased neutrophil and macrophage infiltration into the airspaces of Tg+ mice. These findings are consistent with the studies where inhibition of EGFR signaling increased neutrophil and macrophage recruitment, and increased mRNA expression of chemokines involved in their recruitment to the inflamed tissues (61–65). Consistent with these reports, the Cre^+^/Tg+ mice had increased levels of BALF neutrophil- and macrophage-specific chemokines. Of note, neutrophil elastase (NE), a serine proteinase secreted by neutrophils, has been implicated in the induction of MCM and mucin expression (66, 67). The ablation of NE in Tg+ mice resulted in a significant decrease in MCM, and expression levels of genes associated with mucous cells and mucins secretion, i.e., *Clca1/Gob5*, *Muc5ac*, and *Muc5b* (68). Therefore, it is likely that the neutrophil-derived NE contributed to the increased MCM in Cre^+^/Tg+ mice. Macrophages are reported to secrete IL-13 in response to stimulation with IL-33, an alarmin that is increased in Tg+ mice (39, 69). Since Cre^+^/Tg+ mice have increased macrophage recruitment, it is possible that increased IL-13 levels in Cre^+^/Tg+ mice is attributable to increased IL-13 production by macrophages.

The airway epithelial cell-specific EGFR deficient Tg+ mice, as compared with the EGFR sufficient Tg+ mice, exhibited significantly increased levels of BALF proteins and dsDNA, which suggests the detrimental effect of EGFR deletion on the epithelial integrity in Tg+ mice. Tight junction, adherens junction, and gap junction proteins are essential for maintaining airway epithelial barrier integrity (70, 71). In the current study, the Cre^+^/Tg+ mice exhibited relatively reduced mRNA levels of genes encoding junction proteins. Additionally, the immunohistochemical staining for E-Cadherin showed reduced staining intensity in Cre^+^/Tg+ mice. This finding is consistent with a study that demonstrated decreased cadherin-based cell adhesion upon downregulation of the EGFR pathway (72). However, the role of EGFR in regulating these junction proteins is unclear, with some studies suggesting positive regulation (72), while others suggesting negative regulation (73–76).

The Tg+ mice are prone to spontaneous bacterial infections arising from the aspiration of bacteria of oropharyngeal origin (29). These bacterial infections are usually resolved by 3–4 weeks of age, likely due to the maturation of the immune system in Tg+ juveniles (29). In the current study, the airway epithelial cell-specific deficiency of EGFR resulted in significantly compromised bacterial clearance in Tg+ mice. Since airway epithelial cell-specific EGFR deficiency in Tg+ mice exaggerated the mucus obstruction, the delayed bacterial clearance in these mice was likely caused by the exaggerated mucus obstruction.

A limitation of this study is that it did not investigate how the EGFR deletion in airway epithelial cells in Tg+ juveniles affects the expression of EGFR in other cell types. Additionally, the effect of EGFR deletion in airway epithelial cells on the recruitment of immune cells were only investigated for 4 immune cell populations i.e., macrophages, eosinophils, neutrophils, and lymphocytes. Given the wide repertoire of immune and non-immune cell population, single cell sequencing experiments in the future to investigate the effect of EGFR deletion in airway epithelial cells in Tg+ juvenile on various cell populations are important.

**Figure 8:**
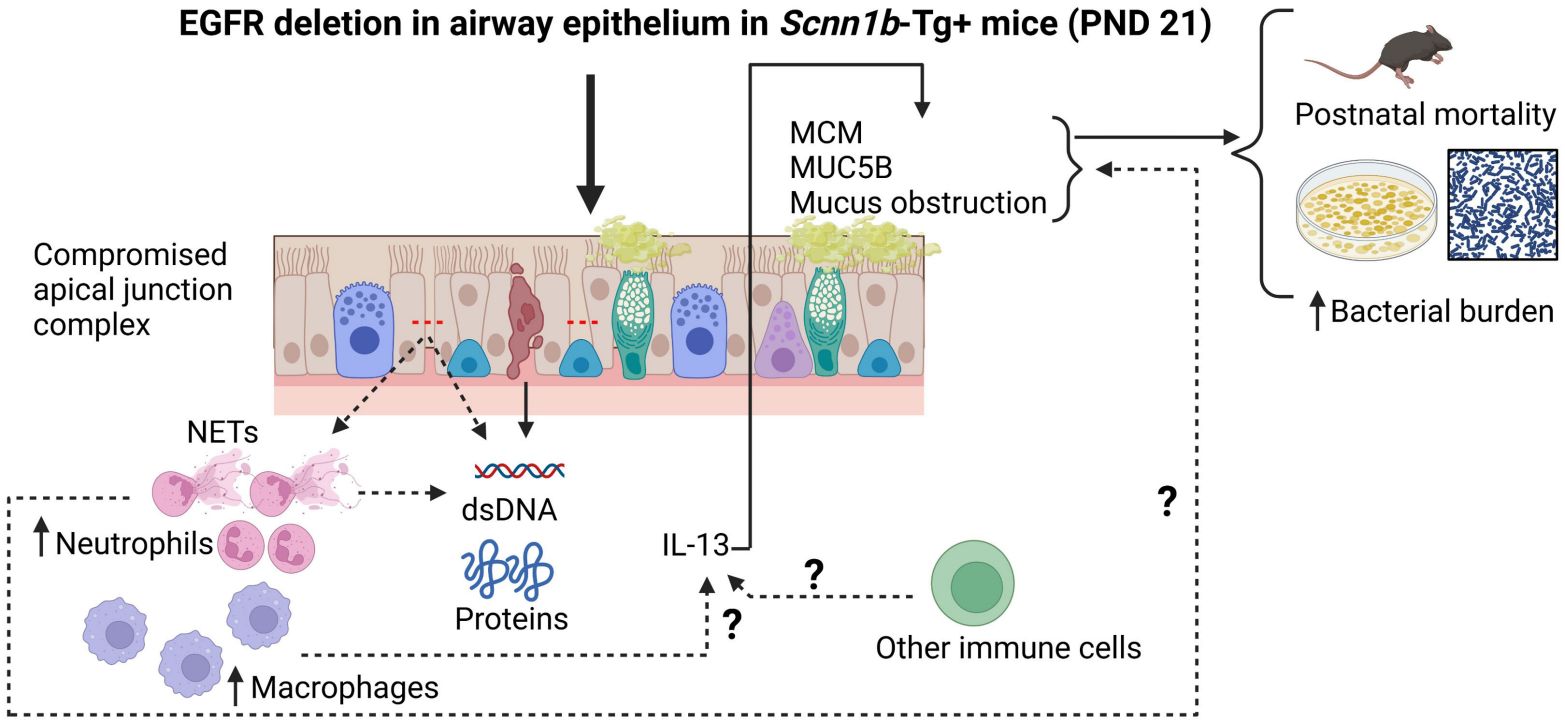
Conceptual model illustrating the potential mechanism by which the absence of EGFR in the airway epithelium increases the mucoinflammatory response in Tg+ mice. The deletion of EGFR in the airway epithelial cells in Tg+ mice triggers a series of events that lead to MCM, MUC5B overproduction and mucus obstruction leading to increased postnatal mortality and increased bacterial burden in these mice. The deletion of EGFR in the airway epithelial cells in Tg+ mice compromises apical junction complex integrity which causes increased recruitment of neutrophils and macrophages in the airway. The compromised epithelial barrier integrity, the NETs and the dying cells contribute to the increased BALF total protein and dsDNA contents. Neutrophil-derived NE, macrophage-derived IL-13 or other immune cell-derived IL-13 may contribute to the MCM, MUC5B overproduction and mucus obstruction leading to postnatal mortality and increased bacterial burden in these mice.

Based on our findings, we propose a conceptual model (**Figure 8**) wherein the deletion of EGFR in the airway epithelial cells in Tg+ mice triggers a series of events that lead to MCM, MUC5B overproduction and mucus obstruction leading to increased postnatal mortality and increased bacterial burden in these mice. The deletion of EGFR in the airway epithelial cells in Tg+ mice compromises apical junction complex integrity, which causes increased recruitment of neutrophils and macrophages in the airway. The compromised epithelial barrier integrity, the NETs and the dying cells contribute to the increased BALF total protein and dsDNA contents. Neutrophil-derived NE, macrophage-derived IL-13 or other immune cell-derived IL-13 may contribute to the MCM, MUC5B overproduction and mucus obstruction leading to postnatal mortality and increased bacterial burden in these mice.

In conclusion, this study demonstrated that the airway epithelial cell-specific deletion of EGFR significantly worsens the mucoinflammatory responses in Tg+ mice lungs, including increased degree of mucus obstruction, MCM, MUC5B production, increased inflammatory cell recruitment, elevated levels of inflammatory mediators and type 2 inflammation-associated markers, compromised postnatal survival, and delayed bacterial clearance. These data highlight the cell-specific role of the EGFR signaling pathway and suggest that the pan-cellular inhibition of this pathway, using pharmacological inhibitors, may worsen mucoinflammatory lung disease.

## Data Availability

The datasets presented in this study can be found in online repositories. The names of the repository/repositories and accession number(s) can be found in the article/**Supplementary Material**.

## Supplementary material

Supplementary Figure 1 Representative photomicrographs of AB-PAS-stained **(A)**, MUC5B-immunostained **(B)**, and MUC5AC-immunostained **(C)** whole left lung lobe sections from Cre^-^/Tg+, and Cre^+^/Tg+ mice. All photomicrographs for each stain across both the groups were taken at the same magnification. (Cre^-^/Tg+ [blue solid border] and Cre^+^/Tg+ [red solid border] mice).

Supplementary Figure 2 Absolute quantification of *Cdh1* mRNA **(A)**, *Cldn5* mRNA **(B)**, *Ctnnb1* mRNA **(C)**, *Ocln* mRNA **(D),** *Tjp1* mRNA **(E**), and *Cx43* mRNA **(F)** in the lungs from WT mice (with Cre^-^ or Cre^+^ status) and Tg+ mice (with Cre^-^ or Cre^+^ status). Sample size (n=5-7/group). **(G)** Representative photomicrographs of E-Cadherin-immunostained left lung lobe sections from Cre^-^/Tg+ and Cre^+^/Tg+ mice. The photomicrographs across both the groups were taken at the same magnification. (Cre^-^/Tg+ [blue solid border] and Cre^+^/Tg+ [red solid border] mice). Blue solid arrows indicate strong E-Cadherin staining in airway epithelial cells while dotted blue arrows indicate reduced staining intensity of E-Cadherin in airway epithelial cells. Error bars represent Mean ± SEM. One-way ANOVA followed by Tukey’s *post hoc* test was used for the statistical analysis. **p* < 0.05. To minimize the number of horizontal lines in various panels (e.g**.,** **Figure 2A**), single horizontal line with small vertical ticks were used to indicate statistically significant differences. The positioning of small vertical ticks indicates the group that is significantly different from the reference group (with no vertical line). In **Figure 2A**, single horizontal significance line with three vertical ticks suggests significant difference for three comparisons, i.e., Cre^+^/Tg+ vs Cre^-^/Tg+, Cre^+^/Tg+ vs Cre^+^/WT, and Cre^+^/Tg+ vs Cre^-^/WT.

## References

[1] Ratjen F Bell SC Rowe SM Goss CH Quittner AL Bush A. Cystic fibrosis. Nat Rev Dis Primers. (2015) 1:15010. doi: 10.1038/nrdp.2015.10 27189798 PMC7041544

[2] Fahy JV Dickey BF. Airway mucus function and dysfunction. N Engl J Med. (2010) 363:2233–47. doi: 10.1056/NEJMra0910061 PMC4048736 21121836

[3] Boucher RC. Airway surface dehydration in cystic fibrosis: pathogenesis and therapy. Annu Rev Med. (2007) 58:157–70. doi: 10.1146/annurev.med.58.071905.105316 17217330

[4] Symmes BA Stefanski AL Magin CM Evans CM. Role of mucins in lung homeostasis: regulated expression and biosynthesis in health and disease. Biochem Soc Trans. (2018) 46:707–19. doi: 10.1042/BST20170455 PMC8359647 29802217

[5] Burgel PR Nadel JA. Roles of epidermal growth factor receptor activation in epithelial cell repair and mucin production in airway epithelium. Thorax. (2004) 59:992–6. doi: 10.1136/thx.2003.018879 PMC1746853 15516478

[6] Takeyama K Dabbagh K Lee HM Agusti C Lausier JA Ueki IF. Epidermal growth factor system regulates mucin production in airways. Proc Natl Acad Sci U S A. (1999) 96:3081–6. doi: 10.1073/pnas.96.6.3081 PMC15898 10077640

[7] Perrais M Pigny P Copin MC Aubert JP Van Seuningen I. Induction of MUC2 and MUC5AC mucins by factors of the epidermal growth factor (EGF) family is mediated by EGF receptor/Ras/Raf/extracellular signal-regulated kinase cascade and Sp1. J Biol Chem. (2002) 277:32258–67. doi: 10.1074/jbc.M204862200 12077147

[8] Choudhary I Vo T Singamsetty D Paudel K Mao Y Lamichhane R. Myeloid cell-specific IL4Rα deletion protects against mixed allergen-induced lung injury in mice. J Immunol. (2022) 208:109.22–.22. doi: 10.4049/jimmunol.208.Supp.109.22

[9] Le Floc’h A Allinne J Nagashima K Scott G Birchard D Asrat S. Dual blockade of IL-4 and IL-13 with dupilumab, an IL-4Ralpha antibody, is required to broadly inhibit type 2 inflammation. Allergy. (2020) 75:1188–204. doi: 10.1111/all.14151 PMC7317958 31838750

[10] Gour N Wills-Karp M. IL-4 and IL-13 signaling in allergic airway disease. Cytokine. (2015) 75:68–78. doi: 10.1016/j.cyto.2015.05.014 26070934 PMC4532591

[11] Livraghi A Grubb BR Hudson EJ Wilkinson KJ Sheehan JK Mall MA. Airway and lung pathology due to mucosal surface dehydration in beta-epithelial Na+ channel-overexpressing mice: role of TNF-alpha and IL-4Ralpha signaling, influence of neonatal development, and limited efficacy of glucocorticoid treatment. J Immunol. (2009) 182:4357–67. doi: 10.4049/jimmunol.0802557 PMC2659461 19299736

[12] Vallath S Hynds RE Succony L Janes SM Giangreco A. Targeting EGFR signaling in chronic lung disease: therapeutic challenges and opportunities. Eur Respir J. (2014) 44:513–22. doi: 10.1183/09031936.00146413 PMC4209854 24435005

[13] Singh B Carpenter G Coffey RJ. EGF receptor ligands: recent advances. F1000Res. (2016) 5:F1000 Faculty Rev-2270. doi: 10.12688/f1000research PMC5017282 27635238

[14] Threadgill DW Dlugosz AA Hansen LA Tennenbaum T Lichti U Yee D. Targeted disruption of mouse EGF receptor: effect of genetic background on mutant phenotype. Science. (1995) 269:230–4. doi: 10.1126/science.7618084 7618084

[15] Miettinen PJ Berger JE Meneses J Phung Y Pedersen RA Werb Z. Epithelial immaturity and multiorgan failure in mice lacking epidermal growth factor receptor. Nature. (1995) 376:337–41. doi: 10.1038/376337a0 7630400

[16] Sibilia M Wagner EF. Strain-dependent epithelial defects in mice lacking the EGF receptor. Science. (1995) 269:234–8. doi: 10.1126/science.7618085 7618085

[17] Plopper CG St George JA Read LC Nishio SJ Weir AJ Edwards L. Acceleration of alveolar type II cell differentiation in fetal rhesus monkey lung by administration of EGF. Am J Physiol. (1992) 262:L313–21. doi: 10.1152/ajplung.1992.262.3.L313 1550255

[18] Miettinen PJ Warburton D Bu D Zhao JS Berger JE Minoo P. Impaired lung branching morphogenesis in the absence of functional EGF receptor. Dev Biol. (1997) 186:224–36. doi: 10.1006/dbio.1997.8593 9205141

[19] Le Cras TD Hardie WD Fagan K Whitsett JA Korfhagen TR. Disrupted pulmonary vascular development and pulmonary hypertension in transgenic mice overexpressing transforming growth factor-alpha. Am J Physiol Lung Cell Mol Physiol. (2003) 285:L1046–54. doi: 10.1152/ajplung.00045.2003 12896876

[20] Yasui S Nagai A Oohira A Iwashita M Konno K. Effects of anti-mouse EGF antiserum on prenatal lung development in fetal mice. Pediatr Pulmonol. (1993) 15:251–6. doi: 10.1002/ppul.1950150412 8385767

[21] Tyner JW Kim EY Ide K Pelletier MR Roswit WT Morton JD. Blocking airway mucous cell metaplasia by inhibiting EGFR antiapoptosis and IL-13 transdifferentiation signals. J Clin Invest. (2006) 116:309–21. doi: 10.1172/JCI25167 PMC1359039 16453019

[22] Takeyama K Jung B Shim JJ Burgel PR Dao-Pick T Ueki IF. Activation of epidermal growth factor receptors is responsible for mucin synthesis induced by cigarette smoke. Am J Physiol Lung Cell Mol Physiol. (2001) 280:L165–72. doi: 10.1152/ajplung.2001.280.1.L165 11133506

[23] Burgel PR Lazarus SC Tam DC Ueki IF Atabai K Birch M. Human eosinophils induce mucin production in airway epithelial cells via epidermal growth factor receptor activation. J Immunol. (2001) 167:5948–54. doi: 10.4049/jimmunol.167.10.5948 11698473

[24] Shim JJ Dabbagh K Ueki IF Dao-Pick T Burgel PR Takeyama K. IL-13 induces mucin production by stimulating epidermal growth factor receptors and by activating neutrophils. Am J Physiol Lung Cell Mol Physiol. (2001) 280:L134–40. doi: 10.1152/ajplung.2001.280.1.L134 11133503

[25] Burgel PR Montani D Danel C Dusser DJ Nadel JA. A morphometric study of mucins and small airway plugging in cystic fibrosis. Thorax. (2007) 62:153–61. doi: 10.1136/thx.2006.062190 PMC2111259 16928707

[26] Mall M Grubb BR Harkema JR O’Neal WK Boucher RC. Increased airway epithelial Na+ absorption produces cystic fibrosis-like lung disease in mice. Nat Med. (2004) 10:487–93. doi: 10.1038/nm1028 15077107

[27] Mall MA Harkema JR Trojanek JB Treis D Livraghi A Schubert S. Development of chronic bronchitis and emphysema in beta-epithelial Na+ channel-overexpressing mice. Am J Respir Crit Care Med. (2008) 177:730–42. doi: 10.1164/rccm.200708-1233OC PMC2277210 18079494

[28] Saini Y Wilkinson KJ Terrell KA Burns KA Livraghi-Butrico A Doerschuk CM. Neonatal pulmonary macrophage depletion coupled to defective mucus clearance increases susceptibility to pneumonia and alters pulmonary immune responses. Am J Respir Cell Mol Biol. (2016) 54:210–21. doi: 10.1165/rcmb.2014-0111OC PMC4821038 26121027

[29] Livraghi-Butrico A Kelly EJ Klem ER Dang H Wolfgang MC Boucher RC. Mucus clearance, MyD88-dependent and MyD88-independent immunity modulate lung susceptibility to spontaneous bacterial infection and inflammation. Mucosal Immunol. (2012) 5:397–408. doi: 10.1038/mi.2012.17 22419116 PMC3377774

[30] Saini Y Dang H Livraghi-Butrico A Kelly EJ Jones LC O’Neal WK. Gene expression in whole lung and pulmonary macrophages reflects the dynamic pathology associated with airway surface dehydration. BMC Genomics. (2014) 15:726. doi: 10.1186/1471-2164-15-726 25204199 PMC4247008

[31] Mao Y Patial S Saini Y. Airway epithelial cell-specific deletion of HMGB1 exaggerates inflammatory responses in mice with muco-obstructive airway disease. Front Immunol. (2022) 13:944772. doi: 10.3389/fimmu.2022.944772 36741411 PMC9892197

[32] Choudhary I Vo T Paudel K Yadav R Mao Y Patial S. Postnatal ozone exposure disrupts alveolar development, exaggerates mucoinflammatory responses, and suppresses bacterial clearance in developing scnn1b-tg(+) mice lungs. J Immunol. (2021) 207:1165–79. doi: 10.4049/jimmunol.2001286 PMC8654340 34330754

[33] Choudhary I Vo T Bathula CS Lamichhane R Lewis BW Looper J. Tristetraprolin overexpression in non-hematopoietic cells protects against acute lung injury in mice. Front Immunol. (2020) 11:2164. doi: 10.3389/fimmu.2020.02164 32983182 PMC7493631

[34] Patial S Lewis BW Vo T Choudhary I Paudel K Mao Y. Myeloid-IL4Ralpha is an indispensable link in IL-33-ILCs-IL-13-IL4Ralpha axis of eosinophil recruitment in murine lungs. Sci Rep. (2021) 11:15465. doi: 10.1038/s41598-021-94843-9 34326406 PMC8322172

[35] Schindelin J Arganda-Carreras I Frise E Kaynig V Longair M Pietzsch T. Fiji: an open-source platform for biological-image analysis. Nat Methods. (2012) 9:676–82. doi: 10.1038/nmeth.2019 PMC3855844 22743772

[36] Choudhary I Vo T Paudel K Patial S Saini Y. Compartment-specific transcriptomics of ozone-exposed murine lungs reveals sex- and cell type-associated perturbations relevant to mucoinflammatory lung diseases. Am J Physiol Lung Cell Mol Physiol. (2021) 320:L99–L125. doi: 10.1152/ajplung.00381.2020 33026818 PMC7847060

[37] Choudhary I Vo T Paudel K Wen X Gupta R Kesimer M. Vesicular and extravesicular protein analyses from the airspaces of ozone-exposed mice revealed signatures associated with mucoinflammatory lung disease. Sci Rep. (2021) 11:23203. doi: 10.1038/s41598-021-02256-5 34853335 PMC8636509

[38] Vo T Paudel K Choudhary I Patial S Saini Y. Ozone exposure upregulates the expression of host susceptibility protein TMPRSS2 to SARS-CoV-2. Sci Rep. (2022) 12:1357. doi: 10.1038/s41598-022-04906-8 35079032 PMC8789794

[39] Lewis BW Vo T Choudhary I Kidder A Bathula C Ehre C. Ablation of IL-33 suppresses th2 responses but is accompanied by sustained mucus obstruction in the scnn1b transgenic mouse model. J Immunol. (2020) 204:1650–60. doi: 10.4049/jimmunol.1900234 PMC7714586 32060135

[40] Lewis BW Choudhary I Paudel K Mao Y Sharma R Wang Y. The innate lymphoid system is a critical player in the manifestation of mucoinflammatory airway disease in mice. J Immunol. (2020) 205:1695–708. doi: 10.4049/jimmunol.2000530 PMC7717108 32817334

[41] Joyner BL Jones SW Cairns BA Harris BD Coverstone AM Abode KA. DNA and inflammatory mediators in bronchoalveolar lavage fluid from children with acute inhalational injuries. J Burn Care Res. (2013) 34:326–33. doi: 10.1097/BCR.0b013e31825d5126 PMC3567221 23128126

[42] Kirchner KK Wagener JS Khan TZ Copenhaver SC Accurso FJ. Increased DNA levels in bronchoalveolar lavage fluid obtained from infants with cystic fibrosis. Am J Respir Crit Care Med. (1996) 154:1426–9. doi: 10.1164/ajrccm.154.5.8912759 8912759

[43] Kwong K Vaishnav RA Liu Y Subhedar N Stromberg AJ Getchell ML. Target ablation-induced regulation of macrophage recruitment into the olfactory epithelium of Mip-1alpha-/- mice and restoration of function by exogenous MIP-1alpha. Physiol Genomics. (2004) 20:73–86. doi: 10.1152/physiolgenomics.00187.2004 15467013

[44] Nath A Chattopadhya S Chattopadhyay U Sharma NK. Macrophage inflammatory protein (MIP)1alpha and MIP1beta differentially regulate release of inflammatory cytokines and generation of tumoricidal monocytes in Malignancy. Cancer Immunol Immunother. (2006) 55:1534–41. doi: 10.1007/s00262-006-0149-3 PMC11030200 16518599

[45] Driscoll KE. Macrophage inflammatory proteins: biology and role in pulmonary inflammation. Exp Lung Res. (1994) 20:473–90. doi: 10.3109/01902149409031733 7882902

[46] Sitkauskiene B Johansson AK Sergejeva S Lundin S Sjostrand M Lotvall J. Regulation of bone marrow and airway CD34+ eosinophils by interleukin-5. Am J Respir Cell Mol Biol. (2004) 30:367–78. doi: 10.1165/rcmb.2002-0311OC 12920051

[47] Rosenberg HF Phipps S Foster PS. Eosinophil trafficking in allergy and asthma. J Allergy Clin Immunol. (2007) 119:1303–10; quiz 11-2. doi: 10.1016/j.jaci.2007.03.048 17481712

[48] Lewis BW Sultana R Sharma R Noel A Langohr I Patial S. Early postnatal secondhand smoke exposure disrupts bacterial clearance and abolishes immune responses in muco-obstructive lung disease. J Immunol. (2017) 199:1170–83. doi: 10.4049/jimmunol.1700144 28667160

[49] Inoue H Akimoto K Homma T Tanaka A Sagara H. Airway epithelial dysfunction in asthma: relevant to epidermal growth factor receptors and airway epithelial cells. J Clin Med. (2020) 9(11):3698. doi: 10.3390/jcm9113698 PMC7698733 33217964

[50] Adib-Conquy M Pedron T Petit-Bertron AF Tabary O Corvol H Jacquot J. Neutrophils in cystic fibrosis display a distinct gene expression pattern. Mol Med. (2008) 14:36–44. doi: 10.2119/2007-00081.Adib-Conquy 18026571 PMC2078559

[51] Birchenough GM Johansson ME Gustafsson JK Bergstrom JH Hansson GC. New developments in goblet cell mucus secretion and function. Mucosal Immunol. (2015) 8:712–9. doi: 10.1038/mi.2015.32 PMC4631840 25872481

[52] Livraghi-Butrico A Grubb BR Wilkinson KJ Volmer AS Burns KA Evans CM. Contribution of mucus concentration and secreted mucins Muc5ac and Muc5b to the pathogenesis of muco-obstructive lung disease. Mucosal Immunol. (2017) 10:395–407. doi: 10.1038/mi.2016.63 27435107 PMC5250616

[53] Hur GY Lee SY Lee SH Kim SJ Lee KJ Jung JY. Potential use of an anticancer drug gefinitib, an EGFR inhibitor, on allergic airway inflammation. Exp Mol Med. (2007) 39:367–75. doi: 10.1038/emm.2007.41 17603291

[54] Tamaoka M Hassan M McGovern T Ramos-Barbon D Jo T Yoshizawa Y. The epidermal growth factor receptor mediates allergic airway remodeling in the rat. Eur Respir J. (2008) 32:1213–23. doi: 10.1183/09031936.00166907 18653647

[55] Le Cras TD Acciani TH Mushaben EM Kramer EL Pastura PA Hardie WD. Epithelial EGF receptor signaling mediates airway hyperreactivity and remodeling in a mouse model of chronic asthma. Am J Physiol Lung Cell Mol Physiol. (2011) 300:L414–21. doi: 10.1152/ajplung.00346.2010 PMC3064289 21224214

[56] Choudhary I Lamichhane R Singamsetty D Vo T Brombacher F Patial S. Cell-specific contribution of IL4 receptor α signaling shapes the overall manifestation of allergic airway disease. Am J Respir Cell Mol Biol (accepted publication). (2024) 71(6):702-17. doi: 10.1165/rcmb.2024-0208OC PMC11622633 39254378

[57] Bruscia EM Bonfield TL. Innate and adaptive immunity in cystic fibrosis. Clin Chest Med. (2016) 37:17–29. doi: 10.1016/j.ccm.2015.11.010 26857765

[58] Sagel SD Kapsner R Osberg I Sontag MK Accurso FJ. Airway inflammation in children with cystic fibrosis and healthy children assessed by sputum induction. Am J Respir Crit Care Med. (2001) 164:1425–31. doi: 10.1164/ajrccm.164.8.2104075 11704590

[59] Elizur A Cannon CL Ferkol TW. Airway inflammation in cystic fibrosis. Chest. (2008) 133:489–95. doi: 10.1378/chest.07-1631 18252915

[60] Saini Y Lewis BW Yu D Dang H Livraghi-Butrico A Del Piero F. Effect of LysM+ macrophage depletion on lung pathology in mice with chronic bronchitis. Physiol Rep. (2018) 6:e13677. doi: 10.14814/phy2.13677 29667749 PMC5904692

[61] Harada C Kawaguchi T Ogata-Suetsugu S Yamada M Hamada N Maeyama T. EGFR tyrosine kinase inhibition worsens acute lung injury in mice with repairing airway epithelium. Am J Respir Crit Care Med. (2011) 183:743–51. doi: 10.1164/rccm.201002-0188OC 20935109

[62] Mascia F Lam G Keith C Garber C Steinberg SM Kohn E. Genetic ablation of epidermal EGFR reveals the dynamic origin of adverse effects of anti-EGFR therapy. Sci Transl Med. (2013) 5:199ra10. doi: 10.1126/scitranslmed.3005773 PMC6324537 23966299

[63] Wang Y Cheng S Zhang H Zhang Y Ding C Peng T. Adverse effects of gefitinib on skin and colon in a lung cancer mouse model. Recent Pat Anticancer Drug Discov. (2024) 19:308–15. doi: 10.2174/1574892818666230727143750 37723963

[64] Bangsgaard N Houtkamp M Schuurhuis DH Parren PW Baadsgaard O Niessen HW. Neutralization of IL-8 prevents the induction of dermatologic adverse events associated with the inhibition of epidermal growth factor receptor. PloS One. (2012) 7:e39706. doi: 10.1371/journal.pone.0039706 22761877 PMC3382563

[65] Dube PE Liu CY Girish N Washington MK Polk DB. Pharmacological activation of epidermal growth factor receptor signaling inhibits colitis-associated cancer in mice. Sci Rep. (2018) 8:9119. doi: 10.1038/s41598-018-27353-w 29904166 PMC6002410

[66] Voynow JA Young LR Wang Y Horger T Rose MC Fischer BM. Neutrophil elastase increases MUC5AC mRNA and protein expression in respiratory epithelial cells. Am J Physiol. (1999) 276:L835–43. doi: 10.1152/ajplung.1999.276.5.L835 10330040

[67] Voynow JA Fischer BM Malarkey DE Burch LH Wong T Longphre M. Neutrophil elastase induces mucus cell metaplasia in mouse lung. Am J Physiol Lung Cell Mol Physiol. (2004) 287:L1293–302. doi: 10.1152/ajplung.00140.2004 15273079

[68] Gehrig S Duerr J Weitnauer M Wagner CJ Graeber SY Schatterny J. Lack of neutrophil elastase reduces inflammation, mucus hypersecretion, and emphysema, but not mucus obstruction, in mice with cystic fibrosis-like lung disease. Am J Respir Crit Care Med. (2014) 189:1082–92. doi: 10.1164/rccm.201311-1932OC 24678594

[69] Yang Z Grinchuk V Urban JF Jr. Bohl J Sun R Notari L. Macrophages as IL-25/IL-33-responsive cells play an important role in the induction of type 2 immunity. PLoS One. (2013) 8:e59441. doi: 10.1371/journal.pone.0059441 23536877 PMC3607614

[70] Yuksel H Ocalan M Yilmaz O. E-cadherin: an important functional molecule at respiratory barrier between defense and dysfunction. Front Physiol. (2021) 12:720227. doi: 10.3389/fphys.2021.720227 34671272 PMC8521047

[71] Freund-Michel V Muller B Marthan R Savineau JP Guibert C. Expression and role of connexin-based gap junctions in pulmonary inflammatory diseases. Pharmacol Ther. (2016) 164:105–19. doi: 10.1016/j.pharmthera.2016.04.004 27126473

[72] Cela C Llimargas M. Egfr is essential for maintaining epithelial integrity during tracheal remodeling in Drosophila. Development. (2006) 133:3115–25. doi: 10.1242/dev.02482 16831830

[73] Petecchia L Sabatini F Usai C Caci E Varesio L Rossi GA. Cytokines induce tight junction disassembly in airway cells via an EGFR-dependent MAPK/ERK1/2-pathway. Lab Invest. (2012) 92:1140–8. doi: 10.1038/labinvest.2012.67 22584669

[74] Gavard J Gutkind JS. A molecular crosstalk between E-cadherin and EGFR signaling networks. In: Haley JD Gullick WJ, editors. EGFR Signaling Networks in Cancer Therapy, vol. p . Humana Press, Totowa, NJ (2008). p. 131–46.

[75] Lo HW Hsu SC Xia W Cao X Shih JY Wei Y. Epidermal growth factor receptor cooperates with signal transducer and activator of transcription 3 to induce epithelial-mesenchymal transition in cancer cells via up-regulation of TWIST gene expression. Cancer Res. (2007) 67:9066–76. doi: 10.1158/0008-5472.CAN-07-0575 PMC2570961 17909010

[76] Ramirez Moreno M Bulgakova NA. The cross-talk between EGFR and E-cadherin. Front Cell Dev Biol. (2021) 9:828673. doi: 10.3389/fcell.2021.828673 35127732 PMC8811214

