# Supplementary figures and images for: Airway epithelial cell-specific deletion of EGFR modulates mucoinflammatory features of cystic fibrosis-like lung disease in mice
Source: Front Immunol. 2025 May 8;16:1493950. doi: 10.3389/fimmu.2025.1493950 (PMC12094982; doi:10.3389/fimmu.2025.1493950)

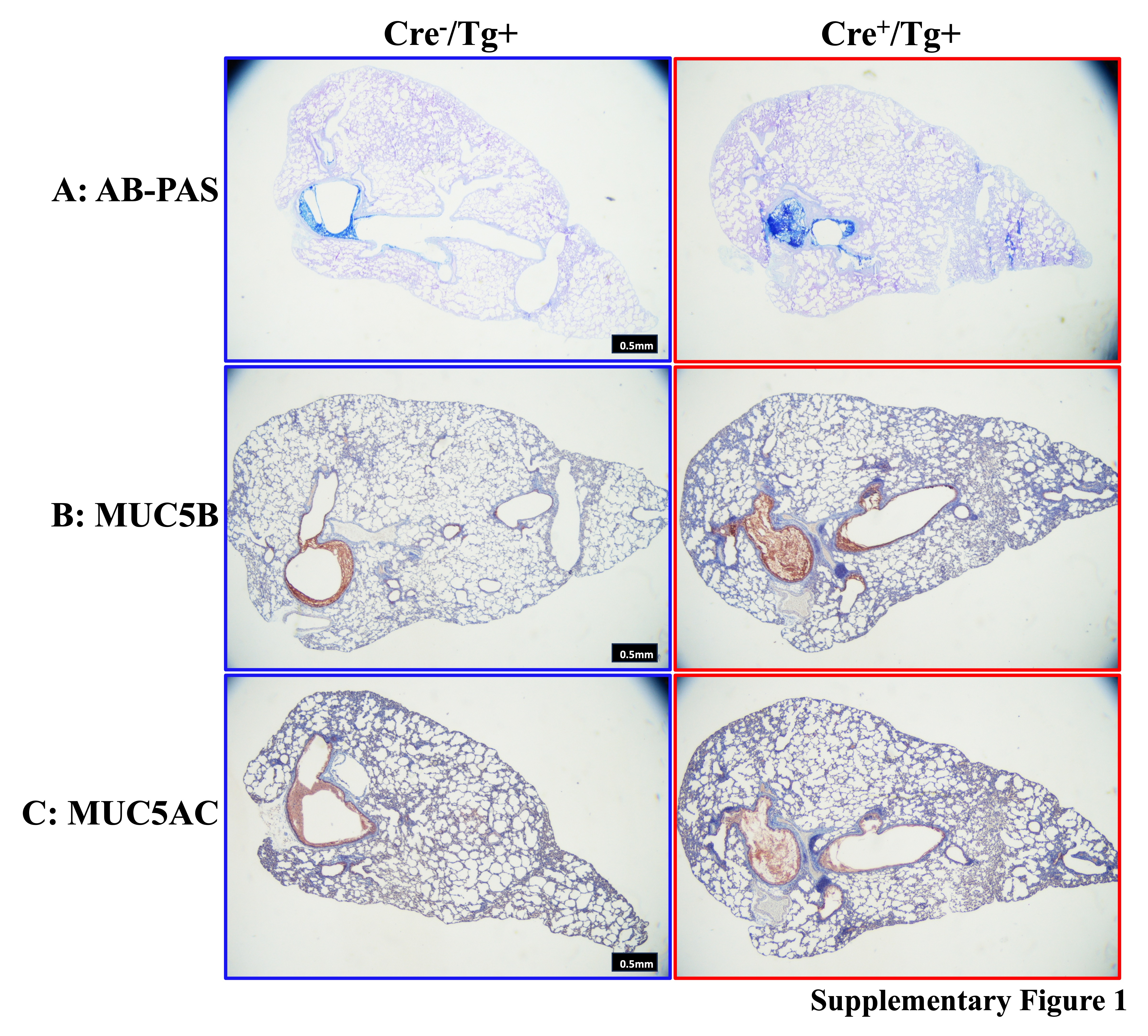

Supplement: Supplementary Figure 1 — Representative photomicrographs of AB-PAS-stained (A), MUC5B-immunostained (B), and MUC5AC-immunostained (C) whole left lung lobe sections from Cre-/Tg+, and Cre+/Tg+ mice. All photomicrographs for each stain across both the groups were taken at the same magnification. (Cre-/Tg+ [blue solid border] and Cre+/Tg+ [red solid border] mice). [file Image1.jpeg]

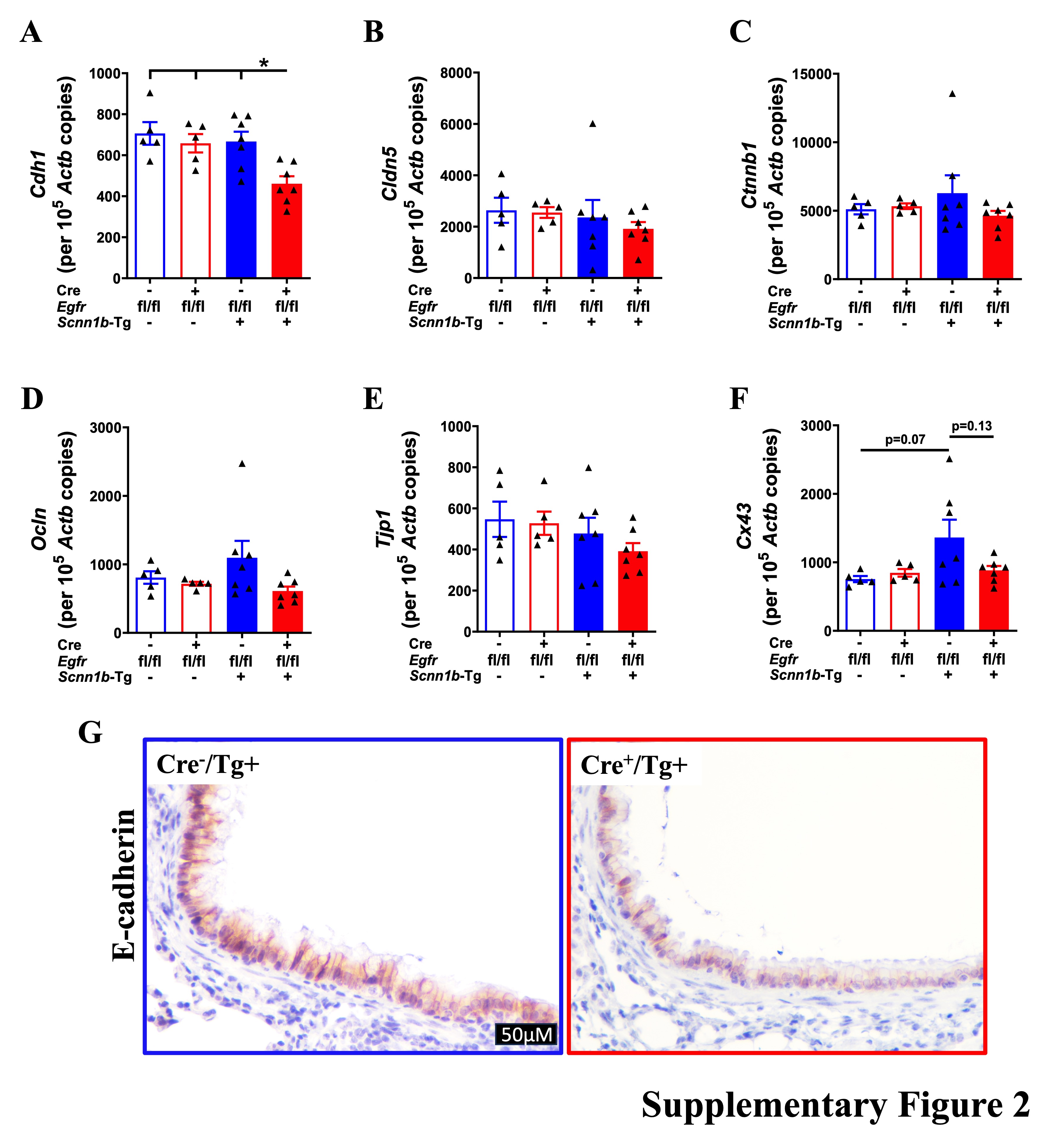

Supplement: Supplementary Figure 2 — Absolute quantification of Cdh1 mRNA (A), Cldn5 mRNA (B), Ctnnb1 mRNA (C), Ocln mRNA (D), Tjp1 mRNA (E), and Cx43 mRNA (F) in the lungs from WT mice (with Cre- or Cre+ status) and Tg+ mice (with Cre- or Cre+ status). Sample size (n=5-7/group). (G) Representative photomicrographs of E-Cadherin-immunostained left lung lobe sections from Cre-/Tg+ and Cre+/Tg+ mice. The photomicrographs across both the groups were taken at the same magnification. (Cre-/Tg+ [blue solid border] and Cre+/Tg+ [red solid border] mice). Blue solid arrows indicate strong E-Cadherin staining in airway epithelial cells while dotted blue arrows indicate reduced staining intensity of E-Cadherin in airway epithelial cells. Error bars represent Mean ± SEM. One-way ANOVA followed by Tukey’s post hoc test was used for the statistical analysis. *p < 0.05. To minimize the number of horizontal lines in various panels (e.g., Figure 2A ), single horizontal line with small vertical ticks were used to indicate statistically significant differences. The positioning of small vertical ticks indicates the group that is significantly different from the reference group (with no vertical line). In Figure 2A , single horizontal significance line with three vertical ticks suggests significant difference for three comparisons, i.e., Cre+/Tg+ vs Cre-/Tg+, Cre+/Tg+ vs Cre+/WT, and Cre+/Tg+ vs Cre-/WT. [file Image2.jpeg]
