# Supplementary material for: Airway epithelial cell-specific deletion of EGFR modulates mucoinflammatory features of cystic fibrosis-like lung disease in mice
Source: Front Immunol. 2025 May 8;16:1493950. doi: 10.3389/fimmu.2025.1493950 (PMC12094982; doi:10.3389/fimmu.2025.1493950)
Supplement: Supplementary file 3 [file DataSheet1.pdf]

**Supplemental Table 1:** Primer sequences for PCR genotyping and RT-qPCR

| No | Gene Symbol | Forward Sequence | Reverse Sequence |
|---|---|---|---|
| **PCR Genotyping Primers** | | | |
| 1 | *Scnn1b* (genomic DNA; *gDNA)* | CTTCCAAGAGTTCAACTACCG | TCTACCAGCTCAGCCACAGTG |
| 2 | *EGFR (gDNA)* | CTGCTACTGGCTCAAGTTTC | CTTTGGAGAACCTGCAGATC |
| 3 | *CC10-Cre (gDNA)* | TCTGATGAAGTCAGGAAGAACC | GAGATGTCCTTCACTCTGATTC |
| 4 | *CC10-Cre Internal Control (gDNA)* | TGCCAGAGATTGTTCTAGAAAACAA | GGCACAATGATGTTAATGACGTAAA |
| **RT-qPCR Primers** | | | |
| 1 | *Actb (mRNA)* | GGCTGTATTCCCCTCCATCG | GGGGTACTTCAGGGTCAGGA |
| 2 | *Il13 (mRNA)* | ATCACACAAGACCAGACTCCC | CTCTGGGTCCTGTAGATGGC |
| 3 | *Cx43 (mRNA)* | ACAGGTCTGAGAGCCCGAAC | GTCTGGGCACCTCTCTTTCAC |
| 4 | *Clca1 (mRNA)* | AAGCAGTGAGGTGTTCAGCA | CAGTCCCGTTACTCTGTCGAT |
| 5 | *Slc26a4 (mRNA)* | GACTGTAAAGACCCTCTTGATCTGA | GGAAGCAAGTCTACGCATGG |
| 6 | *Retnla (mRNA)* | CCTGCTGGGATGACTGCTAC | CAGTGGTCCAGTCAACGAGT |
| 7 | *Cldn5 (mRNA)* | GTTAAGGCACGGGTAGCACT | GTACTTCTGTGACACCGGCA |
| 8 | *Chi3l4 (mRNA)* | CCACTTTGAACCACATTCCAAGG | GAGAGACTGAGACAGTTCAGGG |
| 9 | *Ocln (mRNA)* | AAGTCAACACCTCTGGTGCC | TACCATTGCTGCTGTACCGA |
| 10 | *Tjp1 (mRNA)* | GACGGTGGCGTGAGGAG | CATTGCTGTGCTCTTAGCGG |
| 11 | *Ctnnb1 (mRNA)* | GAATGAAGGCGTGGCAACAT | TGGTCAGCTCGACTGAAAGC |
| 12 | *Cdh1 (mRNA)* | AACCCAAGCACGTATCAGGG | GAGTGTTGGGGGCATCATCA |

**Supplemental Table 2**: Cytokine in BALF (picograms per milliliter)

| **Cytokine** | **Cre$^{-}$/WT** | **Cre$^{+}$/WT** | **Cre$^{-}$/Tg+** | **Cre$^{+}$/Tg+** | **LOD** |
|---|---|---|---|---|---|
| IL-6 | 2.49 ± 0.00* | 2.49 ± 0.00$^{\pi}$ | 4.02 ± 1.09 | 18.49 ± 7.13*$^{\pi}$ | 2.5 |
| IL-5 | 0.83 ± 0.32* | 1.12 ± 0.50$^{\pi}$ | 77.86 ± 18.50*$^{\pi}$ | 40.04 ± 12.35 | 0.37 |
| RANTES | 2.06 ± 0.14 | 1.62 ± 0.15* | 1.16 ± 0.17* | 1.30 ± 0.15 | 0.17 |
| MIP-1α | 8.53 ± 0.86*$^{\Psi}$ | 10.41 ± 2.74$^{\pi\Phi}$ | 62.68 ± 11.85$^{\#\Psi\Phi}$ | 119.2 ± 13.34*$^{\pi\#}$ | 7.68 |
| MIP-1β | 9.69 ± 1.34* | 7.65 ± 0.00$^{\pi}$ | 26.01 ± 4.46$^{\#}$ | 54.34 ± 7.68*$^{\pi\#}$ | 7.66 |
| KC | 3.15 ± 0.37* | 2.08 ± 0.47$^{\pi}$ | 112.60 ± 29.57$^{\#}$ | 226.30 ± 42.87*$^{\pi\#}$ | 0.55 |
| IL-4 | 0.43 ± 0.11*$^{\#}$ | 0.07 ± 0.00$^{\pi\Psi}$ | 12.69 ± 3.37*$^{\pi}$ | 16.44 ± 3.84$^{\#\Psi}$ | 0.08 |
| TNF-α | 1.46 ± 0.55* | 0.77 ± 0.14$^{\pi}$ | 1.23 ± 0.29$^{\#}$ | 4.44 ± 0.77*$^{\pi\#}$ | 0.64 |
| MIP-2 | 50.72 ± 11.50* | 27.09 ± 13.07$^{\pi}$ | 305.40 ± 70.28$^{\#}$ | 894.3 ± 163.3*$^{\pi\#}$ | 14.03 |
| G-CSF | 0.22 ± 0.05* | 0.39 ± 0.14$^{\pi}$ | 200.1 ± 66.41$^{\#}$ | 475.2 ± 76.40*$^{\pi\#}$ | 0.18 |
| GM-CSF | 8.42 ± 0.64 | 9.78 ± 1.57 | 15.35 ± 3.38 | 16.60 ± 3.69 | 7.44 |
| IL-10 | 12.73 ± 6.53*$^{\pi}$ | 4.35 ± 1.43 | 0.99 ± 0.32* | 0.45 ± 0.00$^{\pi}$ | 0.46 |
| MCP-1 | 16.67 ± 4.70 | 5.10 ± 0.00 | 18.20 ± 5.42 | 15.10 ± 5.20 | 5.11 |
| IL-9 | 116.5 ± 16.45 | 86.05 ± 14.84 | 106.20 ± 14.90 | 119.8 ± 10.65 | 23.89 |
| IL-1α | 23.33 ± 8.29 | 18.19 ± 5.33 | 7.43 ± 3.05 | 19.45 ± 5.27 | 1.73 |
| IFN-λ | 3.36 ± 0.96 | 1.03 ± 0.00 | 1.27 ± 0.19 | 2.05 ± 1.01 | 1.04 |
| IP-10 | 4.99 ± 0.58* | 4.81 ± 0.53$^{\pi}$ | 10.30 ± 1.96*$^{\pi}$ | 9.10 ± 1.22 | 1.68 |
| IL-17 | 0.84 ± 0.21 | 0.54 ± 0.25 | 0.86 ± 0.34 | 0.88 ± 0.15 | 0.00 |

Two values sharing identical designations (* or $^{\pi}$ or $^{\#}$ or $^{\Psi}$ or $^{\Phi}$) within a row indicate significant differences. Values that were below the LOD [< out-of-range (OOR)] were assigned value 0.01 unit less than the LOD. n = 7-9 in each group, $p < 0.05$. LOD means lower limit of detection or lowest value obtained in the assay.
